# Syncing sustainable urban mobility with public transit policy trends based on global data analysis

**DOI:** 10.1038/s41598-021-93741-4

**Published:** 2021-07-20

**Authors:** Avishai (Avi) Ceder

**Affiliations:** 1 Civil and Environmental Engineering, Technion-Israel Institute of Technology, Haifa, Israel; 2 Faculty of Engineering, University of Auckland, Auckland, New Zealand; 3 Beijing Jiaotong University, Beijing, China; 4 IDEC Hiroshima University, Hiroshima, Japan

**Keywords:** Environmental sciences, Environmental social sciences

## Abstract

Unforeseeable developments will accompany progressive COVID-19 recovery globally. Similarly, science will inform changes amidst its own progress. Social isolation and distancing imposed by the pandemic are likely to result in changed habits, behavior, and thinking paradigms. Inevitably, this should affect the tremendous confusion inhibiting automated urban mobility's evolution. While mobility often seems magnanimously resistant to change, using international data, this analysis shows road traffic, the largest net contributor to global warming, is responsible for even greater damages. The core claim justifies replacing private cars (PCs) by existing and future public transit (PT) vehicles. In testing 17 major cities globally, 94% of the scenarios proved PT superior or equivalent to PCs for reducing travel time. As a result, a foreseeable, future scenario shows potential reduction in car traffic by approximately two-thirds compared with the current situation. In two arenas, proactive government can promote such sustainable urban mobility: (1) developing autonomous vehicles for PT only; (2) coordinating standardization for seamless urban mobility. These global decisions for improving our lives in the future are likely to be better received and understood subsequent to COVID-19, as the focus of our concerns changes from what preoccupied us under the circumstances prior to the pandemic.

## Introduction

As the world nurses its wounds and recovers from the devastating effects of COVID-19, there is an opportunity to plan and surrender to changes that will result in a healthier, improved world. Moreover, globally, in the post pandemic crisis, people may be more willing to change their thinking paradigm, habits and behavior. This brings us to the observation that rapid progress in technology has resulted in revolutionary development of the Internet and cellular phones, while tremendous confusion^1–6^ continues to inhibit the evolution of automated urban mobility. On the verge of a dramatic change, mobility^7,8^ faces a window of opportunity, yet seems to resist submitting itself to the process, albeit ultramodern, yet similar to the complicated and disconcerting 50-year transition from horse to car^9,10^.

This work is about the opportunities available to us for changing urban mobility, and subsequently, significantly diminishing road traffic damages and their global, interdisciplinary implications for global warming^11,12^ and other negative impact^13–17^. The work addresses issues of (a) how to fight the confusion that continues to inhibit the evolution of automated urban mobility (e.g., the deadly Tesla car crash on 17 April, 2021 in Texas), and (b) how important it currently is to channel transit policy trends, to avoid mistakes amidst this interval of confusion in the transition from traditional to automated-electrical vehicles. The paper has two generalized, main purposes. First, it undertakes a unique analysis, to present a new global picture of the current road vehicle situation for a transition period between ordinary and autonomous vehicles (AVs). Secondly, it aims to use that global view as a trigger for reassessment of the direction of AV development and its sustainability. It strives to trigger proactive behavior by which decision makers will introduce new global decisions.

The core claim of this work is based on new modelling by which global data indicate justification for replacing private cars by a range of existing and future public transit vehicles. Gradually the driving habit^18^, or addiction^19^, must be directed elsewhere. Hereafter, the term, public transit (PT), applies to all future service vehicles for people and goods which move horizontally, vertically, or diagonally, on surface, elevated, or underground. All privately used and owned vehicles are designated as private cars (PCs). In simple terms, the paper advocates for automated PT vehicles which meet transportation needs better than the currently prevalent PC mode.

The work is comprised of four parts: a global (19-country) picture of the damages caused by road traffic, a global (17-city) comparison between PT and PCs in terms of urban travel time, a future road mobility scenario and its global (17-city) implications, and a list of contributions and discussion accompanied by a vision for sustainable urban mobility. The work shows that the combination of the first three parts, each with its new modeling and analysis, streamlines into the result and the distinct message that AV development warrants reappraisal. This is done by the first part of the paper highlighting the global aspect of the problem. Thereafter, the second part shows the significant advantage of PT over PCs. The third part is the result of a foreseeable conclusion of the first two parts in term of an example of implied, potential sustainable urban mobility. Finally, the last part cites contributions and draws conclusions from the preceding three-part study, while incorporating a discussion and a vision with respect to the design of future urban mobility.

Unforeseeable implications of the COVID-19 pandemic for future mobility will be able to rely on automation and be controlled by it. However, it is premature to make hypotheses regarding potential changes in transportation needs as new practices evolve in terms of working-from-home work hours and social distance on public transit. Yet, now is the time to plan for implementing changes, when people are compelled to accept them, and more likely to willingly relinquish conveniences of the past, the familiarity of private transportation, for alternatives preferable to them and society.

There is no doubt that sustainable urban mobility is a worthy mission, particularly considering the prevalent confusion regarding the formation of future urban mobility. Sustainability necessarily involves the capability to stand constantly, and therefore, we ought to consider people's preferences and comfort, on top of understanding our future layout through an integrated perspective^20^ of environment, society, and economics. This notion is an element of the recommendation in the discussion section to take all four components—globalization^21^, personalization^22^, prioritization^23^ and coordination of standardization^24^—into account to ultimately attain seamless and appealing urban mobility^4,25^.

## Road traffic damages

### Description and outcome

**Figure 1:**
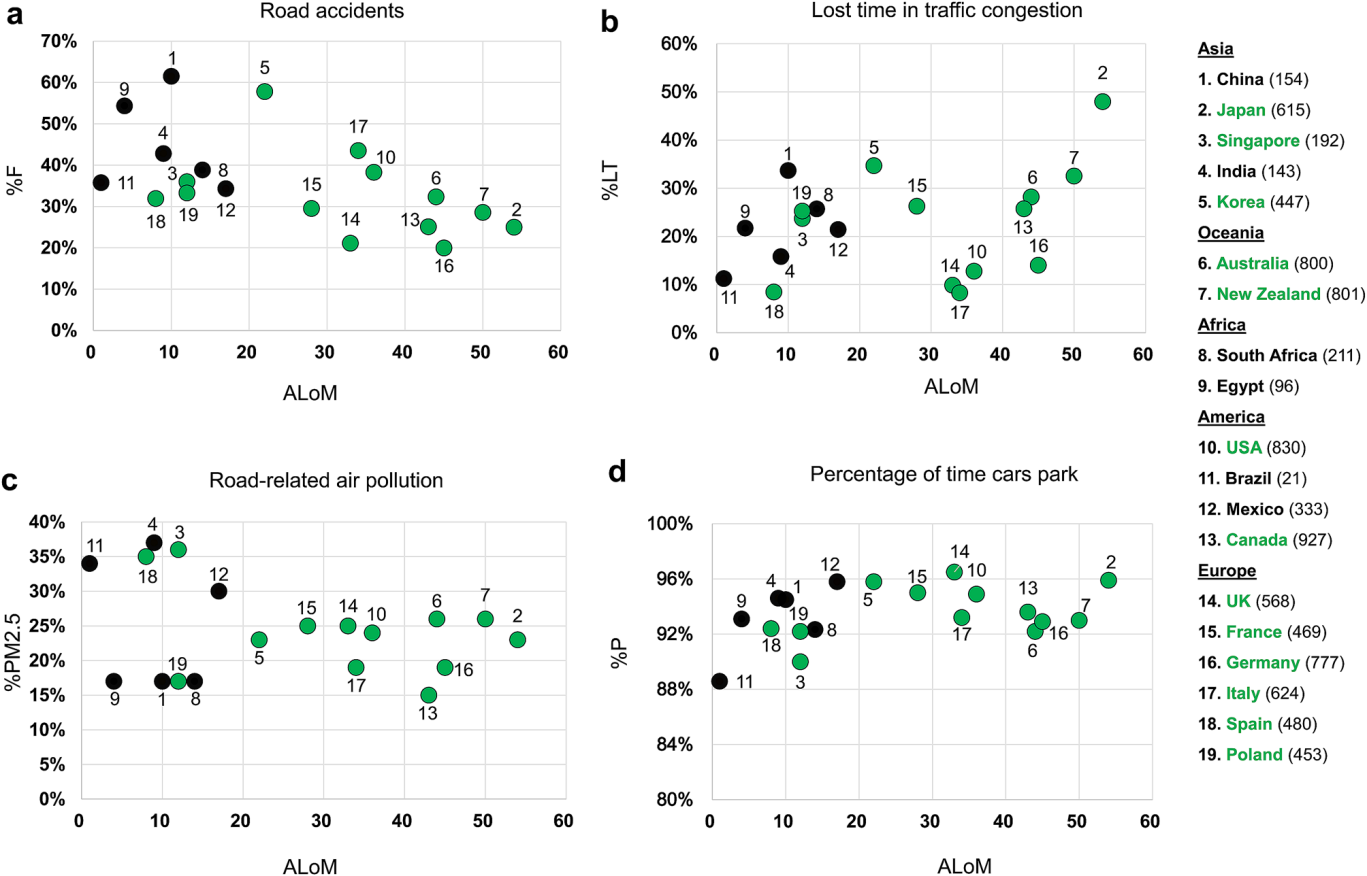
Measures of damages and detrimental impact of road traffic (Supplementary Tables 1 and 8). (**a**) %F is the percentage of road accident fatalities out of the total death rate from accidents of any kind. The ALoM, represents the active level of PC motorization, relates to nonstop (24/7) PCs per 1000 inhabitants. Data points and the list of 19 countries are marked in black for developing countries and green for developed countries. The number in parentheses for each country is the level of PC motorization per 1000 inhabitants, LoM. (**b**) %LT is the percent of lost time in traffic congestion out of total commuting time. (**c**) %PM2.5 is the percent of this type of air pollution from road traffic, from the total %PM2.5 produced. (**d**) %P is the average percentage of time PCs spend parked.

Revolutionary technological developments facilitate progress in AVs, notably providing an opportunity to produce remedies for the damages resulting from PC transportation^13–17^. Fatalities from traffic accidents, time lost in congested traffic, deterioration of public health from automobile-produced air pollution and inefficiency of private cars parked 91–98% of the time (Fig. 1) each assume significant spots in a display case of automobile traffic detriments. Continued reliance on private cars becomes less and less justifiable as the option of automated vehicles continues to evolve.

Road traffic damages represent an unequivocal global problem^13–17,26–30^. For instance, a study by the World Bank and Institute for Health Metrics and Evaluation^14^ shows that the combined global death toll from road crashes and motor vehicle air pollution surpasses global death totals from HIV/AIDS, tuberculosis, malaria, or diabetes, as per Global Burden of Disease data. Although global organizations, such as the World Health Organization (WHO) and the United Nations Road Safety Collaboration (UNRSC), tend to be familiar and concerned with these damages, a concise description of their combined impact has yet to be formulated. Thus, a representative sample of 19 countries demonstrates the magnitude of the problem, showing the detrimental effects of traffic in each case (Fig. 1). The 19 countries were selected to represent two categories: (1) to include countries from all five continents, and (2)—to have a mix of developing and developed countries. An essential factor in this selection process was data availability. The black data points of Fig. 1 are for developing countries and the green points are for developed countries.

In order to address the differences between the extreme measures for each of the different countries, and create a comparison base, four dependent, proportionality-based measures were established. This allows us to exhibit the impact of traffic globally, based on data collected for the year 2014 through 2018. The use of proportionality for the four measures facilitates comprehension of each measure's magnitude. In Fig. 1, the independent measure of active level of PC motorization (ALoM) is equivalent to the number of PCs that run 24-h/day per 1000 inhabitants. This newly calculated ALoM is more representative than the level of PC motorization (LoM) per 1000 inhabitants to show the intensity of PCs producing harmful impact. For example, using the data of Supplementary Table 8 for China, LoM is 217/1.4095 (using millions) = 153.955, such that approximately 154 PCs per 1000 inhabitants are, on the average, running only 6.5% of the time, which is equivalent to 154·6.5/100 = 10 PCs (per 1000 people) running nonstop and generating traffic damages. Certainly, there are many independent variables and measures, but for our purposes, ALoM is a good indicator of the aforementioned intensity.

In Fig. 1, the road accident measure, %F, is the percentage of road accident fatalities of the total death count for the top 50, non-health related causes of accidental death (references shown in Supplementary Table 8). The lost time measure, %LT, is the percent from total commuting time of time lost in traffic congestion. The measure, %PM2.5, is a sampling of air pollution from road traffic, from the total %PM2.5 produced—a dangerous, ambient, particulate matter (PM) with a diameter of less than 2.5 micrometers^17^. The PC park time measure, %P, shows the inefficiency of using PCs, creating unnecessarily occupied urban space, which could potentially serve other essential, public functions. Further explanations appear in “Method” of this section.

In addition to these four negative effects, notably, the transport sector produces almost 25% of total end use energy emissions. The road traffic subsector of this is the largest contributor to global warming or temperature increases for the long term (20–100 years)^11,31^. In light of increased road traffic for passengers and freight, energy related emissions may increase by 50% if no measures are taken^11^ to alleviate the matter.

The problems presented in Fig. 1 are enormous, and can hardly be justified by economic growth and social and public connectivity produced by the transport sector^32^. In effect, road-accident fatalities are responsible for 35.6% of all deaths from accidents (44.6% for developing countries and 31.5% for developed countries). Likewise, lost time is 22.5% of the total commuting time, 24.4% of the total risk PM2.5 comes from road traffic, and 94.7% of the time that PCs spend parked demonstrates their highly inefficient use (Supplementary Tables 1 and 8).

### Method

The objective of this part of the paper is to devise a concise description of the combined impact of road traffic damages. The input data for the four measures of Fig. 1 are exhibited in Supplementary Table 1, and its expanded section in Supplementary Table 8. Regression lines do not appear in Fig. 1 because their R-squared is low; i.e., R-squared of 0.315, 0.001, 0.092, and 0.132 for Fig. 1a–d, respectively. However, the new measures were constructed and are displayed in Fig. 1, in such a way as to reduce the differences between the extreme data for some of the 19 countries. This allows us to compare one country to another and portray the magnitude of road traffic damages.

The calculations of the four measures for each country, are based on: %F = 100·*N*_*f*_*/N*_*d*_ where *N*_*f*_ is the annual number of traffic accident fatalities and *N*_*d*_ is the annual number of deaths from the top 50 non-health related causes of human accidents, %LT = 100·*T*_*l*_*/T*_*c*_ where *T*_*l*_ is the annual number of hours lost in road traffic congestion and *T*_*c*_ is the annual number of hours spent in road traffic commuting, and %PM2.5 = 100·*PM2.5*_*R*_*/PM2.5*_*T*_ where *PM2.5*_*R*_ is the annual amount of PM2.5 produced only by road traffic and *PM2.5*_*T*_ is the total annual amount of PM2.5 produced.

The calculation of %P is based on 100 minus the average percent of PC mobility time in each country. Accordingly, it can be stated that the average annual PC mobility in hours is *T*_pc_ = *D*_*pc*_*/V*_*pc*_ where *D*_*pc*_ is the average PC annual driving distance, and *V*_*pc*_ is the average annual PC road traffic speed. While there are sources for finding the data for *D*_*pc*_ (Supplementary Table 8), *V*_*pc*_ needs to be estimated. Following previous studies^33,34^ the speed distribution is assumed to be uniformly distributed with the attainment of its 50th percentile based on its 85th percentile, where the latter is found from speed limit information^35^. With uniform distribution (UD), the speed *x* is a continuous, random variable with a probability density function of

$$f(x) = \left\{ {\begin{array}{*{20}l} {\frac{1}{{b - a}},} \hfill & {a < x < b} \hfill \\ {0,} \hfill & {else} \hfill \\ \end{array} } \right.$$

where *x* obeys UD for the (*a, b*) section. The parameter *b*–*a* can be derived on the basis of *P*(*x* = 85%) based on the speed limit, and consequently finding *P*(*x* = 50%). Practically speaking, and due to lack of data, it has been determined that the speed limit within towns^35^ may represent the overall average speed of urban and rural areas including cases of traffic congestion. Thus, the speed limit (SL) within towns for the US is 80 km/h^35,36^, with an average for both urban and rural areas of approximately 86 km/h^36^. The calculation of *P*(*x* = 85%) for SL = 80, and SL = 86 results in 94.12, and 101.18, respectively, based on 1/(*b *− *a*). The implications are that *P*(*x* = 50%) will be 47 and 51 km/h, for SL = 80 and SL = 86, respectively. Considering an annual average of 18,000 km per PC in the US, and an annual average of 42 h lost in traffic congestion per PC (Supplementary Tables 1 and 8), the annual average driving hours will be 18,000/47 = 383 h and 18,000/51 = 353 h, for SL = 80 and SL = 86, respectively. However, if the 42 h lost (mostly observed in urban areas) are added to 353 h, one comes close to the 383 h using SL = 80, and thus the estimated *V*_*pc*_ is based on SL within towns^35^. Finally, the calculation of ALoM is based on %P: ALoM = LoM·(100 − %P)/100 to express the level of activity at which PCs generate harmful impacts.

## Transit vs. private cars

### Explanation and outcome

**Figure 2:**
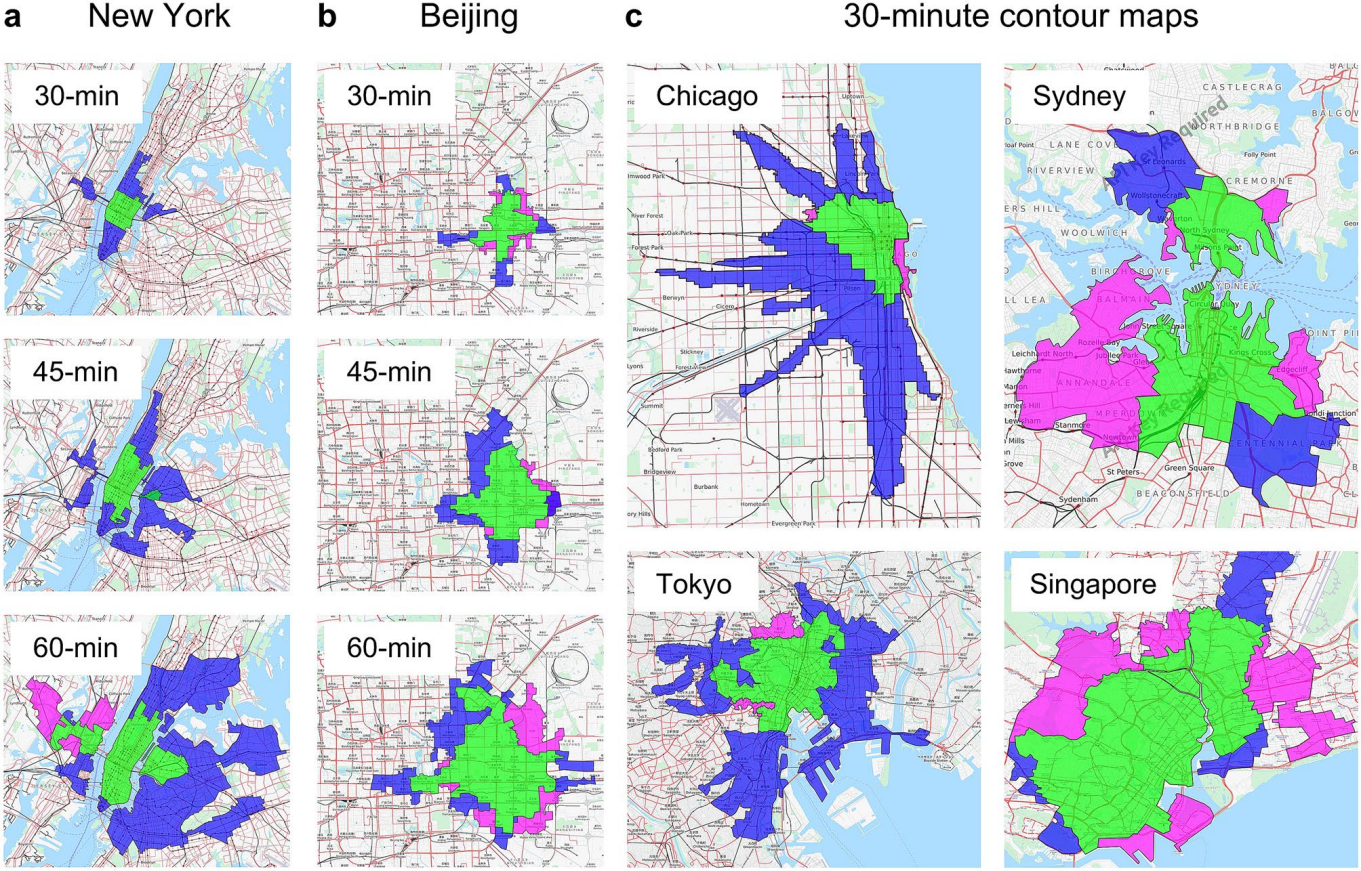
Contour maps of fastest or equal driving time between PT and PCs (Supplementary Tables 2, 3 and 4)**.** PCs are faster than PT (represented in pink); PT is faster than PCs (represented in blue); and the case of equal travel time (represented in green). (**a**) Contour maps for the Lower Manhattan zone of New York showing the fastest modes for reaching destinations within 30, 45, and 60 min between 5:00 and 6:00 p.m. from midtown. (**b**) Contour maps, similar to (**a**), for Guomao zone of Beijing between 5:00 and 6:00 PM from Yintai Center. (**c**) Two strong and two poor PT-related cities in comparison to PC travel times using 30-min contour maps between 5:00 and 6:00 p.m. The selected starting points are from Chicago’s CBD area, Tokyo’s Bunkyo, Sydney’s CBD area, and Singapore’s city center.

**Figure 3:**
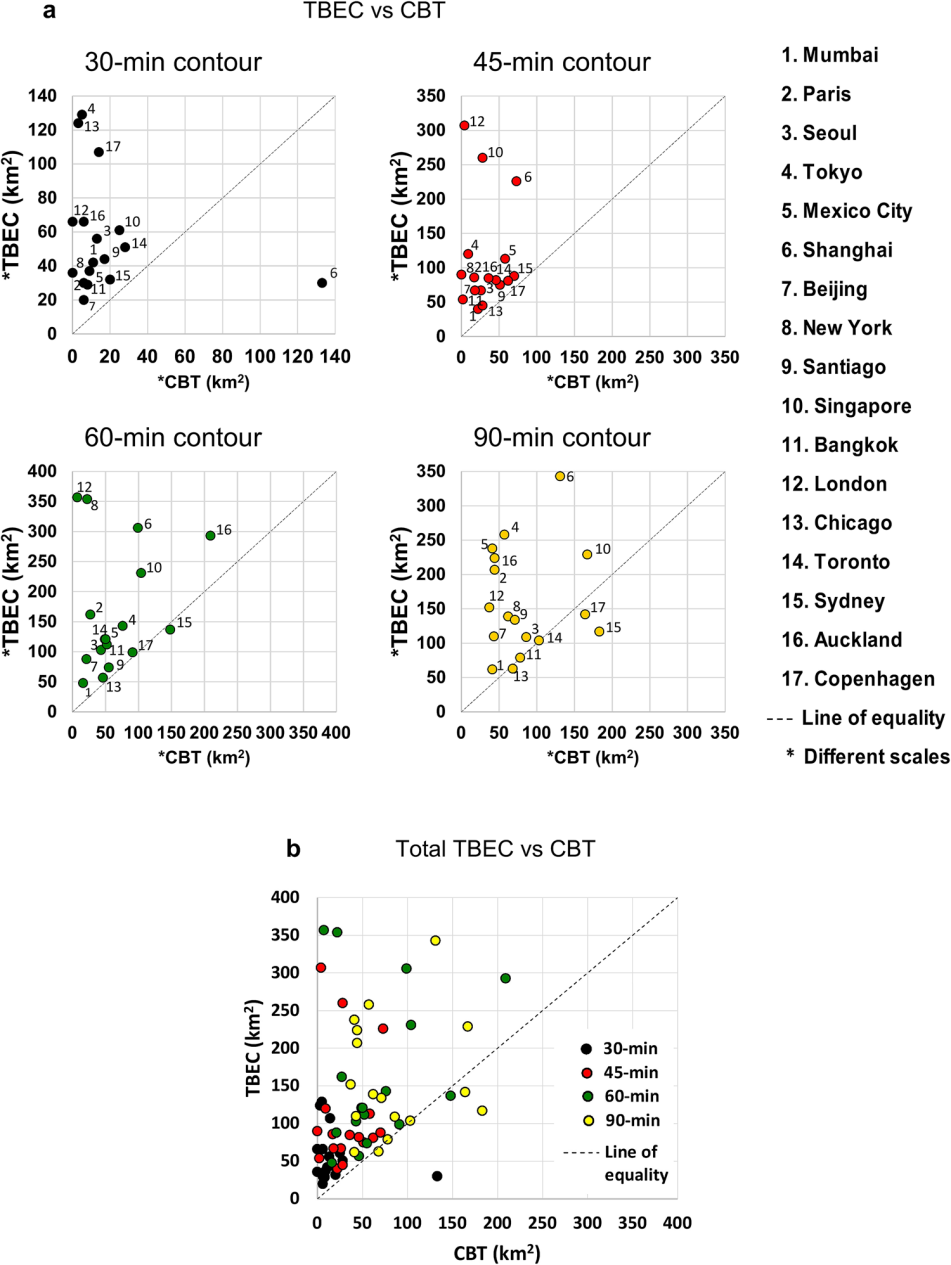
Comparison of fastest travel mode between PT and PCs for 17 cities (Supplementary Tables 2, 3 and 4). (**a**) Data points of the 17 cities for four contour maps, where, in referring to Fig. 2, TBEC (Transit Beats or is Equal to Cars) is the sum of the blue and green areas (in km^2^)**,** CBT (Cars Beat Transit) is the pink area (in km^2^), with city number marked as a data point. The four figures are proportional to the line of equality with different scales used for TBEC and CBT. (**b**) All data points of the four contour-map scenarios for (**a**) using the same colors as for the (**a**) data points.

A comparison of PC performance, the source of the problems, and PT is proposed in light of a move to enlist drastic measures appropriate to the magnitude of these problems and in an attempt to resolve them. A breakthrough towards solving these problems would result from a reasonable reduction or elimination of PCs, compared to the results, yet to be seen from progress to date, primarily with respect to improving traffic safety and reducing traffic generated air pollution by electrification, a consequence of automated technologies. In Figs. 2 and 3, PT is compared to PCs using PM peak data, where all trips start between 5:00 and 6:00 p.m., for 2012–2017, from 17 large cities around the world with urban characteristics appearing in Supplementary Tables 2 and 3. The selection criteria for the 17 cities required that the cities have a popularly known capital and/or mega city across developing and developed countries, with data availability also a necessary consideration. Figures 2 and 3 are a representation of results based on real traffic data applying a methodology developed using newly constructed procedures of ArcGIS 10.3.1, as shown in Supplementary Fig. 1. The compared PT vs. PC performance in Fig. 2 is presented by maximum travel time contour maps showing areas where PT is faster than PCs (blue), PCs are faster than PT (pink), or where their travel times are equal (green). These contour maps refer to a maximum travel time from a chosen point located in the city's center. Figure 3 depicts this comparison for the 17 cities selected, using three acronyms relating to travel times: CBT, cars are better (faster) than transit, or Cars Beat Transit; TBC, transit is better than cars, or Transit Beats Cars; and TBEC, transit is better than or equal to cars, or Transit Beats or is Equal to Cars. The four figures in Fig. 3 correspond to the line of equality.

Supplementary Table 4 shows the results for the 17 cities, for four contour maps of travel times from city centers to each selected zone. The contour map shows T minutes, T = 30, 45, 60, 90, representing how far one can drive by PT or PC in a maximum of T minutes, in all travel directions during PM peak period from a selected city’s center point. The 17 cities and four contour maps generated 68 different scenarios. Figure 2a for the Lower Manhattan zone of New York, and Fig. 2b for the Guomao zone of Beijing show where TBC, CBT and TBEC apply. PC and PT travel times are considered equal if their difference is less than 5%. Using 30-min contour maps, Fig. 2c shows examples of two cities (Chicago and Tokyo) with good PT, and two cities (Sydney and Singapore) with poor PT. For km^2^, TBEC, TBC, CBT values are 124, 103, 3 and 129, 90, 5 for Chicago and Tokyo, respectively, and 61, 17, 25 and 32, 9, 20 for Singapore and Sydney, respectively. The initial point of departure in each city was in the Randolph/Wabash vicinity in Chicago; the Tokyo train station; Westfield, Sydney; and Nex Mall in Singapore.

However, TBC (transit beats cars), TBEC (transit beats or is equal to cars), and CBT (cars beat transit) take more than travel time into account^37–39^. PT route preferences also depend on other attributes including quality of service, fares, accessibility, connectivity and journey distances^40^. Travelers strive for personalized mobility^22,41^ with the option of changing the relative value of TBC and CBT attributes at any time during the day. PT will prevail over PC as a conscious decision if the public will consider it a better fit for its personalized mobility needs. Travel time, however, is a critical attribute, currently^39^ and for the future^42^, in paving the way to considering other attributes.

The methodology behind Figs. 2 and 3, using ArcGIS 10.3.1, is based on the Dijkstra, Prim and Kruskal algorithms for traversing a street network using real time travel speed (Supplementary Fig. 1). Geological street maps are used for network input. The polygons are generated using a triangular irregular network (TIN) format. See “Method” of this section for more information. Finally, Fig. 3a illustrates the data points for the 17 cities, including listing them by their numbers for each of the four contour maps. Figure 3b sums up the four figures of Fig. 3a. Thus, areas in which the PCs are faster on the average than PT, CBT (km^2^) > TBEC (km^2^), were only found for four scenarios: Shanghai for T = 30, Sydney for T = 60, 90, and Copenhagen for T = 90. Overall, PT is superior or equivalent to PCs, entailing less travel time in 94% of the scenarios tested. This represents a significant new finding.

### Method

The objective of this part of the paper is to design a methodology to make global comparisons between average urban travel times for PT and PCs. Data characteristics for Fig. 2 appear in Supplementary Tables 1 and 2, with input and sources of data in Supplementary Table 9. The purpose of Figs. 2 and 3 is to examine which mode of travel is faster, PCs or PT, in the 17 world cities chosen. Four radiuses of travel times from a city center were selected: 30, 45, 60 and 90 min using real data from the street network and travel speed from 5:00 to 6:00 p.m. The methodology for attaining the results in Figs. 2 and 3 is based on constructed procedures of ArcGIS 10.3.1, depicted conceptually in Supplementary Fig. 1; the results are shown in Supplementary Table 4. Basically, two network layers of the PT and PCs are analyzed.

Geologic street maps such as Google Maps, OpenStreetMap, Baidu Maps and NAVTEQ are input as nodes and links, and the Dijkstra, Prim and Kruskal shortest path algorithms create the minimum time-based spanning trees from the selected city’s starting point. All points that are less than or equal to the designated radius travel time are saved for creating the contour map area.

The shortest path routing in terms of travel time is the base of comparison as is shown by Supplementary Fig. 1. For the PCs, different, real observed speeds are used per segment of roadway between two junctions. For the PT, the observed data refer to the timetables and speeds of the available PT travel modes for each end point including the possibility of using more than one PT mode through making transfers. Thereafter, the minimum travel time across all PT modes is selected for comparison with the same for a PC.

Accordingly, for the calculation of PT travel time, all PT routes are examined including bus, metro, rail, and passenger ferry with transfer possibilities across the PT travel modes. Walking time to and from PT stops is based on an average of 5 km/h walking speed. Transfer times, practically speaking, are calculated based on artificial links created between the arrival point of one PT line and the departure point of the other PT line using the average walking speed, where the two PT lines can be of same or different travel mode. The waiting time is derived from the frequency of each PT line based on its timetable assuming that it is half of the headway between two consecutive PT vehicles. The headway is the inverse of the frequency. The use of real data makes it unnecessary to use approximation modelling such as transit assignment models. The complete sources of the input data for ArcGIS 10.3.1 appear in Supplementary Table 9.

## An example of a foreseeable future scenario

### Description and results

Following the global outlook for enormous traffic-damage problems, and the significant (94%) advantage of PT over PCs, we can present an example of potential, sustainable urban mobility as a foreseeable conclusion.

**Figure 4:**
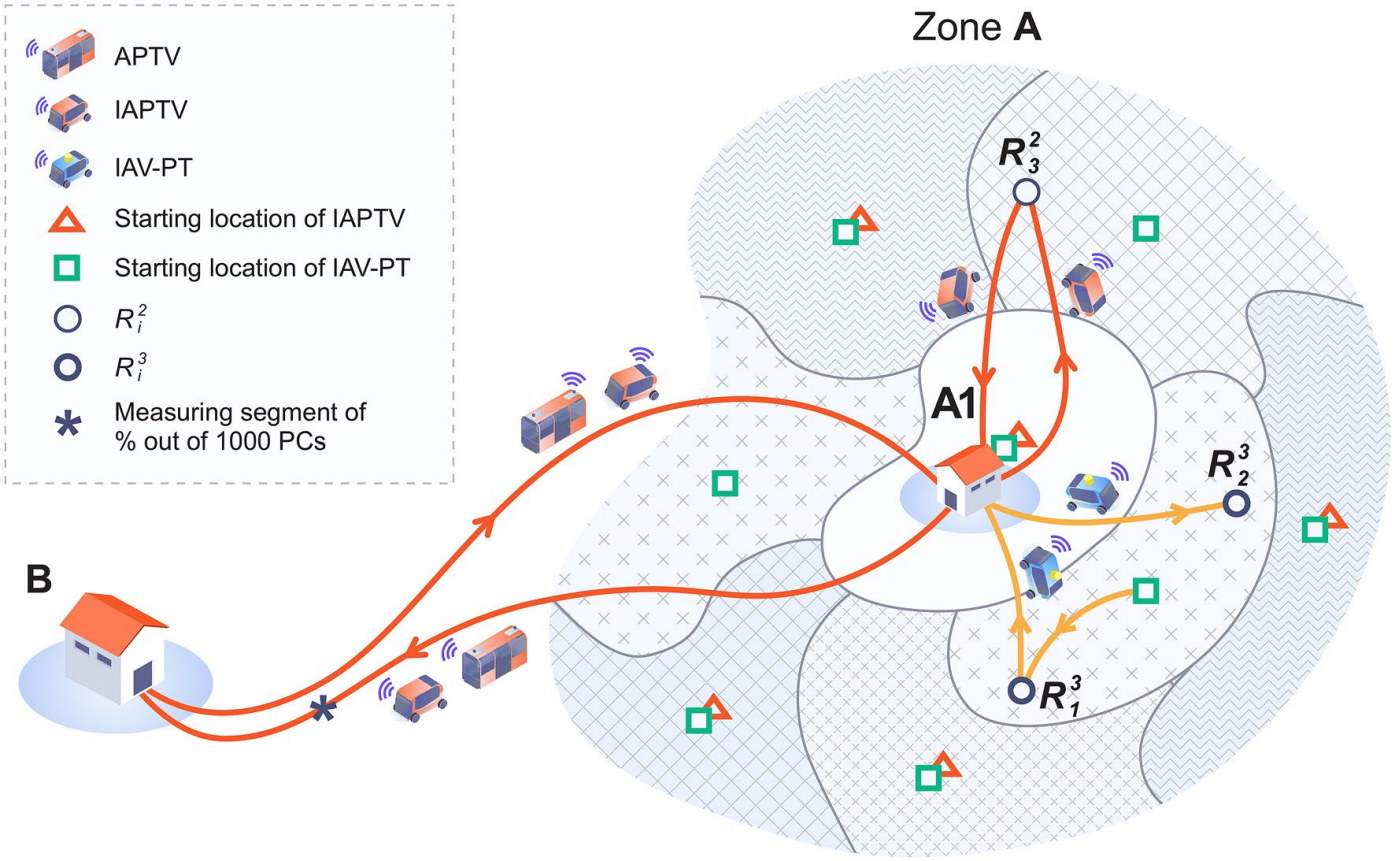
A schematic description of the elements involved with the future mobility scenario**.** It shows that Zone A is comprised of eight TIN sub-areas for individual automated vehicles of local PT service (IAV-PTs), with its eight starting points. However, in this scheme, there are only five starting points for individual automated PT vehicles (IAPTVs) located in five (out of eight) of the IAV-PT starting points. The requests for travel are represented by ${R}_{i}^{2}$ and ${R}_{i}^{3}$ for using IAPTV and IAV-PT, respectively, and there is a measuring point between A1 and B to count the number of shared, automated PT vehicles (APTVs) and IAPTVs compared with the ordinary 1000 PCs.

Thus, an identical, hypothetical, future scenario for all of the 17 cities and its implications is examined in order to derive the number of automated vehicles expected to run in peak hours on the main roads. In constructing this scenario, known notions and research studies on automated vehicles were considered^42–44^. This future scenario is shown schematically in Fig. 4, where Zone A is defined as a residential zone and point B is defined as a real, central shopping point in the canter of each city. The assumption is that a demand, uniformly distributed, of 1000 PCs, each with single person occupancy, is traveling from A to B between 3:00 and 5:00 p.m., using real PC travel time for each city dependent on traffic conditions and road infrastructure. This future scenario exercise examines the benefits and results of shifting the 1000 individuals, driving from A to B using 1000 ordinary PCs, to automated PT vehicles.

As a basis for comparison, travel times from A1 (a central point of A for each of the 17 cities) to B are similar at approximately 30 min, with approximately 20 min travel times in the absence of traffic congestion. Figure 4 assumes automated vehicles, not privately owned, hence considered as PT service, yet the infrastructure is unchanged. Each of the 1000 persons has two choices: (1) to travel from home in A to B using individual automated PT vehicle (IAPTV), or (2) to travel from home (suggested that this be without charge) by individual automated vehicle of local PT service (IAV-PT) to A1 and transfer to shared automated PT vehicle (APTV) that will stop at B.

The methodology developed is based on newly constructed procedures of ArcGIS 10.3.1 shown in Supplementary Fig. 2. In ArcGIS 10.3.1, the shortest path routing (SPR) algorithms are used to create the triangulated irregular network (TIN) area reachable by the maximum time criterion for the movements of IAPTVs and IAV-PTs within zone A, and for APTVs and IAPTVs between A1 and B in both directions. Zone A contains eight TIN sub-areas, SPR-based, with eight starting points for IAV-PTs, but with only five starting points for IAPTVs without showing their TIN sub-areas. An illustrative example is shown in Fig. 4 where a specific IAPTV, with its starting point at A1, is assigned to pick up the ${R}_{3}^{2}$ request, and a specific IAV-PT is assigned to pick up ${R}_{1}^{3}$, drop it off at A1, and then, to handle the ${R}_{2}^{3}$ request without returning to its starting point. In addition, Fig. 4 shows the location of measuring the percent, considering 1000 PCs, of APTVs and IAPTVs for comparing it with the ordinary situation of each of the 17 cities.

**Figure 5:**
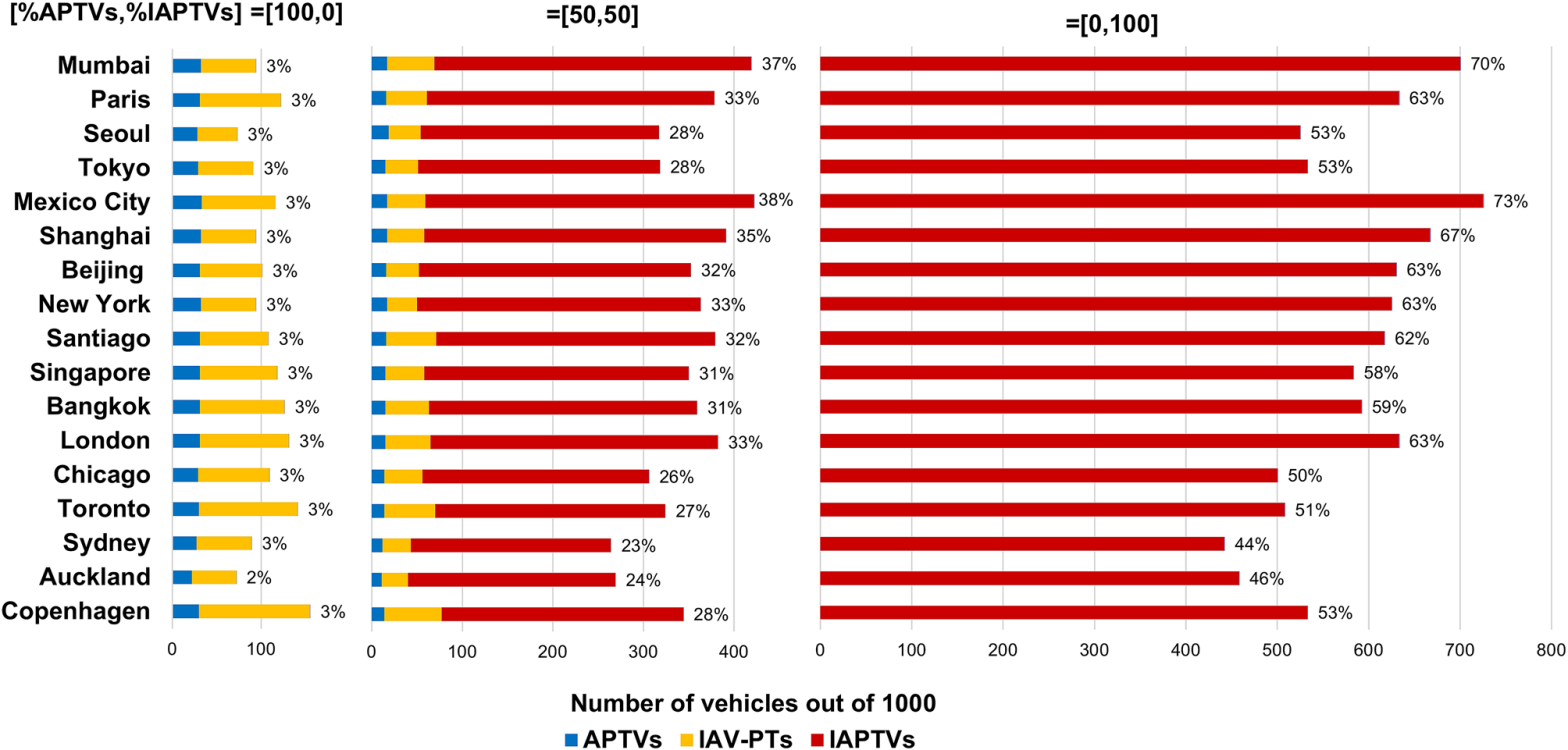
Reduction expected in the number of vehicles based on a future scenario (Fig. 4 and Supplementary Table 6)**.** There are three types of automated vehicles represented: IAPTV, local service IAV-PT, and a shared APTV for three cases: [%APTVs, %IATPVs] = [100, 0], [50, 50] and [0, 100]. The percent at the end of each bar shows the percentage of vehicles on the main road between A and B out of the 1000 ordinary PCs, i.e., without IAV-PTs.

An initial departure location for each person is randomly assigned within A, with maximum eight-minute wait times, and maximum eight-minute ride times by IAV-PTs. APTV and IAPTV speed are assumed to be identical to the speed of a current bus route in a priority lane. APTVs and IAPTVs return to A after completing their trips to B to transport more individuals. Figure 5 shows proportional relations between the percentage of persons using choice (1), and those using choice (2). The methodology related to Fig. 5 appears in “Method” of this section and Supplementary Fig. 2 using ArcGIS 10.3.1. In Fig. 5, there are three cases: [%APTVs, %IATPVs] = [100, 0], [50, 50] and [0, 100] indicating the percentage of people using each of these two modes of travel. Two more cases of [75, 25] and [25, 75] are shown in Supplementary Table 7. The seat occupancy and capacity of an APTV is 20. The IAPTV and IAV-PT can take individuals, groups, a family or other defined group such as friends; however, in this exercise each of these two travel modes takes only one person. The fare for IAPTV will be higher than for a shared APTV. The results of the percentage of automated vehicles on the main road between A and B, out of the 1000 ordinary PCs, appear at the end of each bar.

It is interesting to observe, in this simple exercise, that with only APTVs, the number of vehicles running on the main road between A to B (Fig. 4) will be 3% of the current number of PCs. On the average, this 3% rises to 30% and 58% for the [50, 50] and [0, 100] cases, respectively. The potential reduction in the number of vehicles following their automation corresponds well with the notion that *less is more*. Only the use of PT vehicles has a high chance^45,46^ of reducing the number of vehicles, and thereby contributing to diminishing the negative effects of road traffic.

### Method

The objective of this part is to produce and describe a methodology for global analysis of a future, automated scenario of urban mobility and the expected number of peak-hour vehicles to be operating on the main roads. The schematic sketch of the future scenario examined, appears in Fig. 4 with its 17 city data for A, A1, B shown in Supplementary Table 5. This future scenario exercise investigates the results of a shift of 1000 individuals, going from A to B by 1000 ordinary PCs with single person occupancy, to automated PT vehicles. The methodology for simulating the three layers of APTVs (automated PT vehicles), IAPTVs (individual automated PT vehicles) and IAV-PTs (individual automated vehicles of local PT service) is conceptually depicted in Supplementary Fig. 2. The shortest path routing (SPR) procedures based on Dijkstra, Prim and Kruskal algorithms are used by ArcGIS 10.3.1 to create the triangulated irregular network (TIN) area reachable by the maximum time criterion for the movements of IAPTVs and IAV-PTs within zone A, and for APTVs and IAPTVs from A1 to B and vice versa. Real time traffic data from the 17 cities are used (Supplementary Table 9) to select the A areas and B points to provide a basis of comparison for travel times of approximately 30 min from A to B.

For the same scenario, let *k* represent PT travel mode, where *k* = 1,2,3 applies to APTVs, IAPTVs, and IAV-PTs, respectively. Let *SPR*_*k*_ (*u*,*v*) be SPR (shortest path routing) from *u* to *v* using travel mode(s) *k*,* k* = 1,2,3; and let ${R}_{r}^{k}$ be a travel request made at the point of origin of the PC, *r*, *r* = 1,2,…1000, for travel mode *k*, *k* = 2,3, where all origins are uniformly distributed within A, but their travel requests are made randomly. There are a total of 1000 riding requests between A and B using either choice (1) to travel from home in A to B using IAPTV, or choice (2) to travel from home to A1 using IAV-PT, and from A1 to B using APTV, between 3:00 and 5:00 p.m. in zone A of each city. It is assumed that by using choice (1), IAPTVs are moving to B via A1, and the same applies for the return trip from B to A1. This allows for the use of ArcGIS for this simulated scenario, partially integrated with the APTV movements between A1 and B. Stated in other terms, the pickup for a travel request at *r*, ${R}_{r}^{2}$ will traverse *SPR*_*2*_(*r*,A1) and *SPR*_*2*_(A1*,*B). On the return trip, it will traverse *SPR*_*2*_(B,A1) and *SPR*_*2*_(A1*,q*), where *q* can either be an assigned IAPTV starting point or the location of a new request ${R}_{q}^{2}$.

The assigned IAPTV starting locations and the number of them depend on four parameters: (a) the assumed number of requests (of 1000) for *k* = 2; (b) average round trip time, i.e., *r*–A1–B–A1–*q* using the aforementioned interpretation; (c) maximum pickup time criterion needed to create the TIN area by ArcGIS; and (d) to overlap with an existing (if any) IAV-PT starting location near the center of the TIN area. This is derived from an admin-practical perspective. These assigned locations in the 17 A zones were found iteratively in a manner that follows the concept of gradient-descent^47^ in its simulation of input of the four parameters. The main constraint imposed by this procedure is in assuring that the travel time, i.e., *r*–A1–B, is less than that of an average PC *r*–B travel time.

Individuals who choose (2) will use a transfer between IAV-PT (suggested to be a free ride) and APTV at A1. Thus, their travel time from *r* to B will involve four time elements: waiting time at *r* following ${R}_{r}^{3}$, travel time for *SPR*_*3*_(*r*,A1), waiting time for an APTV departure, and travel time for *SPR*_*1*_(A1,B). The IAV-PTs will also take a returning individual from A1 back to the individual’s assumed home at *r.* The selection of the starting points of IAV-PTs, and the number of them, depend on three parameters: (a) the assumed number of requests (of 1000) for *k* = 1, (b) maximum pickup time following a request, and (c) maximum travel time to A1. The selected starting locations of the 17 A zones, similar to those of the IAPTVs, were found by an iteration process using simulation with an input of the three parameters. The principal constraint in this process is accommodating the *r*–A1–B travel time so that it will be less than for an ordinary PC considering all four of the process's time elements. Certainly, these iterative IAPTV and IAV-PT procedures, for this simple future scenario, beg further elaboration.

Finally, this analysis determines the required number of APTVs, IAPTVs, and IAV-PTs shown in Fig. 5, and in Supplementary Tables 6 and 7. This also implies the number of APTVs and IAPTVs vehicles running during the two hours on the A–B segment. This is interpreted as the percent of automated PT vehicles of 1000 ordinary PCs, as shown in Fig. 5 and cited in Fig. 4. The number of required APTVs is the maximum number of departures during a time window of the round trip time (A1–B–A1)^48,49^. This maximum number of departures is checked throughout the total set of departures simulated over the two hours using the following process: individual passengers arriving randomly at A1 by IAV-PTs and create a departure of an APTV if either the 20-seat occupancy is full, or if the APTV wait time reaches its maximum time. The number of APTV users is given, as well as the number of IAPTV users, and both equal 1000.

The number of required IAPTVs is determined by simulating roundtrip travel time between two travel requests, ${R}_{p}^{2}$ and ${R}_{q}^{2}$, where ${R}_{q}^{2}$ is the first request, feasible to accommodate, after a completion of a roundtrip to B. The implications are that an IAPTV* picks up the individual behind ${R}_{p}^{2}$ who traverses *p*–A1–B–A1–${S}_{l}^{2}$, where ${S}_{l}^{2}$ is its starting location at A, *k* = 2, and traverses ${S}_{l}^{2}$*–q* if the *q* location is within the assigned TIN area of IAPTV*, and there is no other IAPTV that can reach *q* before IAPTV*. This simulation process complies with the constraint of maximum pickup time from making the random travel request for *k* = 2. The number of required IAV-PTs is also determined by simulation, given the number of random requests in the two hours for APTVs, and the starting locations ${S}_{l}^{3}$ for each TIN area selected for IAV-PTs. In this simulation-based procedure, an IAV-PT, after a passenger disembarks at A1, can either traverse its ${S}_{l}^{3}$ or move directly from A1 to a new travel request, as is shown by Fig. 4.

## Contributions and discussion

The contributions of this work are the findings of (1) the magnitude of the global, urban superiority of PT vehicles over PCs (94%), (2) the magnitude of the global picture, and types of road traffic damages, (3) the prospects for reducing the number of vehicles in urban areas using automation by approximately two-thirds, and (4) the unique construction of this three-part study that streamlines into the discussion and conclusions of the overall work. The first three contributions are an assemblage of new concepts and data analyses that have not heretofore been considered in this way*.* These contributions are the fundamental input for the following discussion and vision.

The limitations of the study are prescribed by the attempt to cover the big picture as required for drawing conclusions about policy trends, diminishing possibilities for referring to possible fluctuations of data, and its sensitivity analysis. Thus, the methodologies developed for attaining the contour maps and creating a future mobility scenario (Supplementary Figs. 1 and 2) do not include stochastic optimization with consideration of randomness.

With the current COVID-19 pandemic climacteric, there is a window of opportunity for governments proactively find ways to reduce progressively increasing road traffic damages with the urban mobility of the future. This presumption is integral to this paper from the introduction through to its conclusions. In the ordinary flow of society and social lives, conservative attitudes natural to people, feeling safe in their familiar routines, inhibit change in our best interests. In the context of this work, that refers to eliminating private cars as a means of transportation.

Colossal road traffic damages (for 19-countries) are shown globally and contrary to sustainability, using four traffic-related considerations: road-accident fatalities as responsible for 35.6% of all deaths from accidents; time lost in traffic congestion as 22.5% of total commuting time; 24.4% of total risk PM2.5 coming from road traffic; and 94.7% of the time that PCs spend parked indicative of their highly inefficient use.

Part of the misunderstanding regarding future mobility has its source in the vast amount of emerging terminologies. PT can be perceived as an umbrella over all non-PCs, including those of transportation network companies (TNCs)^5,50^ and mobility-as-a-service (MaaS), as well as transportation of goods^51^. Currently, most future mobility news is about technological advances, along with research and modelling of AVs in consideration of operational and economic issues^52,53^, human and societal perspectives^1–3,6,19,38,44,54^, and government interference^43,55^. However, while technological progress will continue, the most dramatic, anticipated change will be the cessation of driving PCs. The question is how to reach that point wisely.

Driving practices should change^3^, and be perceived, perhaps, like the process of quitting smoking^56^. It involves changing people’s habits^18^ and confronting a sort of addiction to driving^19^. In a study of applied psychology^57^ it is shown that people often do not behave in a manner corresponding to their intentions because of various difficulties and/or temptations. Schwarzer^57^ suggests the use of self‐efficacy and strategic planning in understanding the inconsistency between intention and behavior. This angle seems to be useful for any habit (not only health-compromising behavior). Thus, the intention to use AVs is not easily implemented because of health- and habit-compromising behaviors.

One tactic to reduce PC driving is to provide incentives complemented by clearly demonstrating that transit is better than cars for individuals and society. This should be comprehended by individuals and groups as a choice and not as a stipulated measure. Additionally, the implications of PC disappearance including the fate of PC vehicles with the discontinuation of their use and a range of employment ramifications require attention.

## Vision and concluding remarks

**Figure 6:**
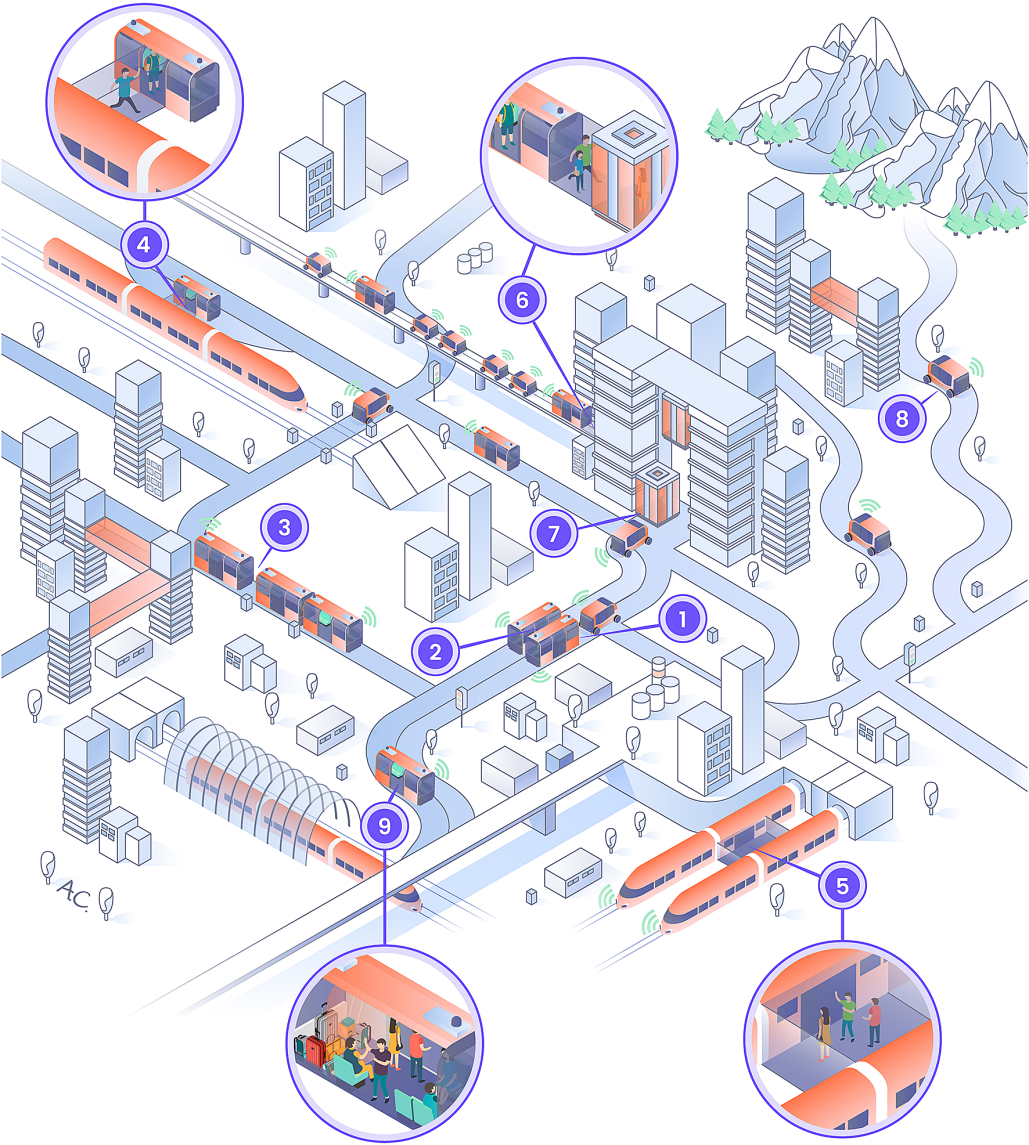
A vision of PT-based seamless urban future mobility. This is a schematic illustration of a feasible urban scenario allowing vehicles to connect to each other physically for smooth transfer of travelers, based on GPPS. Seamless connections of nine vehicles are represented: (1) a longitudinal connection between IAPTV (individual automated PT vehicle) and a small APTV (automated PT vehicle), (2) a lateral connection between two small APTVs, (3) a longitudinal connection between small and large APTVs, (4) a lateral connection between high speed rail and a small APTV, (5) a lateral connection between city and intercity rail, (6) a longitudinal connection between a vertical/diagonal elevator and a small APTV, (7) a longitudinal connection between a horizontal and vertical elevator and a small APTV, (8) a IAPTV journey outside city region, and (9) the flexibility of each APTV to carry goods and freight.

The preferred choice of using public transit over cars bears potential for the realization of sustainable, future, urban mobility. Figure 6, as a vision, shows an appealing future concept for urban mobility without PCs, based on globalization, personalization, prioritization, and standardization (GPPS) with special standardization requirements. Globalization is for reciprocal action between urban and national economies^21^; personalization is for riding preference requests using two-way apps^22,41^; prioritization is for emergency transport, elderly people, VIP with higher service classes^23^, etc. and standardization is for maximizing compatibility of connecting any two PT vehicles^24^. By analogy, standardization is unlike the lack of a universal electric plug because of various countries' preferences for developing their own. One important feature in making PT better than cars is to combine its seamless mobility^25,48^ with travelers’ preferences^22^. PT travelers are likely to have preferences depending on different attributes such as travel time, comfort, fares, accessibility, and connectivity. These can vary, for instance, by time of day, mood, schedule to follow, family considerations, etc. Accordingly, a combined system of seamless mobility with human preferences can be based on a well-designed smartphone application interacting with the PT operator in terms of sending preferences, receiving tailored information, and optimally adjusting system operation and synchronization^48^.

The seamless connections shown by Fig. 6, based on GPPS, demonstrate a future automated transit system centrally synchronized to meet people’s best real time preference-based requests. It illustrates a mobility system moving horizontally and vertically, and possibly diagonally. The synchronized, seamless connections, between and within IAPTVs (individual automated PT vehicles) and APTVs (automated PT vehicles), can be made either laterally (connections 2, 4 and 5 in Fig. 6) or longitudinally (connections 1, 3, 6 and 7). Figure 6 includes an example (number 8) of a journey outside the urban area where further coordinated connections can take place^58^. Finally, it provides one example (number 9) of the use of an APTV to deliver goods/freight that either belong to the travelers or are an additional service taking advantage of the automated system. Certainly, the logistics of such an ideal and sustainable future mobility must be based on prudent algorithms, real time processing with a strong backup, and securely protected procedures. It will also include optimal allocation of the location and parking of idle, automated vehicles. These logistics will follow a period of gradual changes incorporated with some new ideas^59^ for this term.

A perception of a future PT service similar to services of a supermarket is behind Fig. 6. By this conceptualization, ordering food is analogous to IAPTVs, while visiting a supermarket and collecting merchandise from a broad variety of choices is analogous to economy, premium, first class APTVs. Ultimately, this study aims to trigger a process of rethinking the unclear course of future-mobility development, by being proactive and allowing AVs only for standardized PT, using the present window of opportunity. With recent changes in perception of social distance, resulting from the COVID-19 pandemic, this study should contribute to inevitable popular and scholarly processes of rethinking concomitant with changing habits, behavior, and thinking paradigms, and to accepting new, automated, healthier, improved means of urban mobility.

## Supplementary Information

Supplementary Information.
