# Author Correction: Syncing sustainable urban mobility with public transit policy trends based on global data analysis

**DOI:** 10.1038/s41598-021-98446-2

**Published:** 2021-09-15

**Authors:** Avishai (Avi) Ceder

**Affiliations:** 1 Civil and Environmental Engineering, Technion-Israel Institute of Technology, Haifa, Israel; 2 Faculty of Engineering, University of Auckland, Auckland, New Zealand; 3 Beijing Jiaotong University, Beijing, China; 4 IDEC Hiroshima University, Hiroshima, Japan

Correction to: *Scientific Reports* 10.1038/s41598-021-93741-4, published online 20 July 2021

The original version of this Article contained a repeated error in the % of the total risk of PM2.5.

In the section “Road traffic damages”,

“Likewise, lost time is 22.5% of the total commuting time, 14.4% of the total risk PM2.5 comes from road traffic, and 94.7% of the time that PCs spend parked demonstrates their highly inefficient use (Supplementary Tables 1 and 8).”

now reads:

“Likewise, lost time is 22.5% of the total commuting time, 24.4% of the total risk PM2.5 comes from road traffic, and 94.7% of the time that PCs spend parked demonstrates their highly inefficient use (Supplementary Tables 1 and 8).”

In the section, “Contributions and discussion”,

“Colossal road traffic damages (for 19-countries) are shown globally and contrary to sustainability, using four traffic-related considerations: road-accident fatalities as responsible for 35.6% of all deaths from accidents; time lost in traffic congestion as 22.5% of total commuting time; 14.4% of total risk PM2.5 coming from road traffic; and 94.7% of the time that PCs spend parked indicative of their highly inefficient use.”

now reads:

“Colossal road traffic damages (for 19-countries) are shown globally and contrary to sustainability, using four traffic-related considerations: road-accident fatalities as responsible for 35.6% of all deaths from accidents; time lost in traffic congestion as 22.5% of total commuting time; 24.4% of total risk PM2.5 coming from road traffic; and 94.7% of the time that PCs spend parked indicative of their highly inefficient use.”

The original Article has been corrected.

