# Supplementary material for: Syncing sustainable urban mobility with public transit policy trends based on global data analysis
Source: Sci Rep. 2021 Jul 20;11:14597. doi: 10.1038/s41598-021-93741-4 (PMC8292461; doi:10.1038/s41598-021-93741-4)
Supplement: Supplementary file 1 — Supplementary Information. [file 41598_2021_93741_MOESM1_ESM.pdf]

Scientific Reports

Supplementary Information for

Syncing sustainable urban mobility with public transit policy trends based on global data analysis

Avishai (Avi) Ceder, Civil and Environmental Engineering, Technion-Israel Institute of Technology, Haifa, Israel; also, at Faculty of Engineering, University of Auckland, New Zealand; Beijing Jiaotong University, Beijing, China; and IDEC Hiroshima University, Japan. Email: ceder@technion.ac.il , Tel: +972-50-5216084.

This part includes:

Supplementary Fig. 1. A conceptual flowchart for attaining contour maps.
Supplementary Fig. 2. A conceptual flowchart for creating a future mobility scenario.

Supplementary Table 1. Data and results of road traffic damages.
Supplementary Table 2. Characteristics and data, part 1, of areas presented by Figs. 2 and 3.
Supplementary Table 3. Characteristics and data, part 2, of areas presented by Figs. 2 and 3.
Supplementary Table 4. Results of contour areas for four travel times used in Figs. 2 and 3.
Supplementary Table 5. Characteristics of A, A1, and B of the 17-city.
Supplementary Table 6. Number of automated vehicles out of 1000 PCs for three cases represented in Fig. 5.
Supplementary Table 7. Number of automated vehicles out of 1000 PCs for two additional cases.
Supplementary Table 8. Information for Supplementary Table 1 with a list of references/sources indicated by superscripts.
Supplementary Table 9. Information on data sources and input data for ArcGIS 10.3.1.

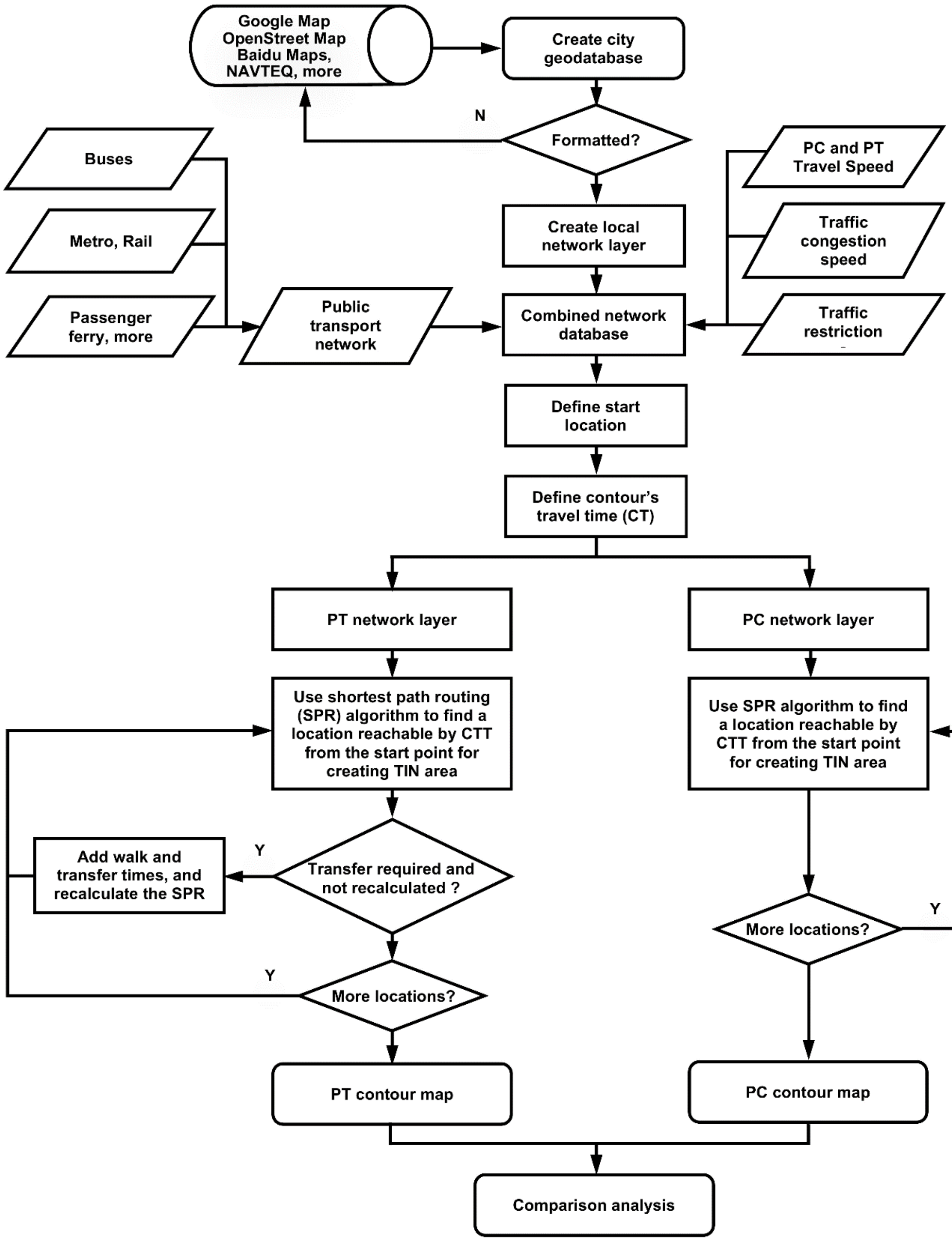

Supplementary Fig. 1. A conceptual flowchart for attaining contour maps. The process occurring using ArcGIS 10.3.1 to create the PC and PT transport network layers followed by the SPR algorithm to all locations feasible to reach within, or at the maximum, travel time chosen of 30, 45, 60 or 90 minutes. This generates the PC, PT and the overlapped TIN area for each, for all of the 17 cities shown in Figs. 2 and 3 using actual input data.

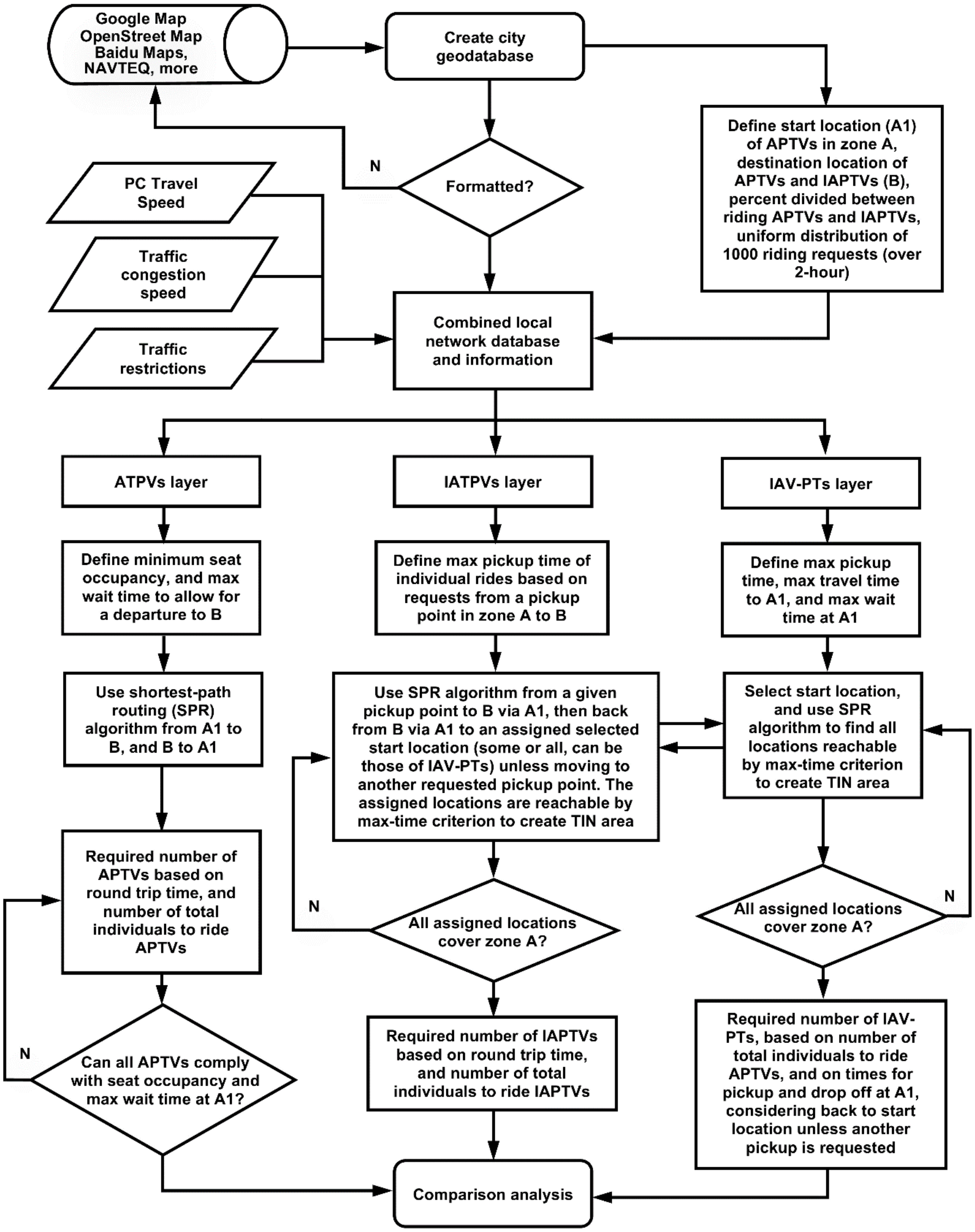

Supplementary Fig. 2. A conceptual flowchart for creating a future mobility scenario. The construction of the APTVs, IAPTVs, and IAV-PTs transport network layers and the general procedures for pickup, drop off, and traversing SPR based sections of 1000 individual travel requests. It shows the criteria of the analysis and the use of ArcGIS 10.3.1 for creating firstly, a small TIN area around the starting points of IAPTVs, and IAV-PTs, and secondly, the TIN area of the entire A zone. Actual data input were used to determine the number of automated vehicles required, as shown in Fig. 5.

Supplementary Table 1. Data and results of road traffic damages

Group	Country	D_{pc} (km)	V_{pc} (km/hr)	LT_{PC} (hr/year)	%LT	%F	%PM 2.5	%P	Pop (M)	# PCs (M)	ALoM
**Asia**	China	15,000	26	80	33.68	61.49	17	93.5	1,409.5	217.0	10
	Japan	18,000	24	78	48.00	13.99	23	91.3	127.5	78.1	54
	Singapore	16,700	29	87	23.73	33.77	36	93.5	5.7	1.0	12
	India	20,000	35	60	15.82	42.82	37	93.5	1,339.2	191.0	9
	Korea	13,651	32	107	34.70	57.81	23	95.2	51.0	22.8	22
**Oceania**	Australia	15,530	32	94	28.20	32.33	26	94.5	24.5	19.2	44
	New Zealand	10,400	24	80	32.54	28.57	26	95.0	4.7	3.8	50
**Africa**	South Africa	20,000	35	90	25.71	38.84	17	93.5	56.7	12.0	14
	Egypt	14,000	35	76	21.71	54.35	17	95.5	97.6	9.4	4
**America**	USA	18,000	47	42	12.76	38.30	24	95.6	324.5	268.8	36
	Brazil	12,000	29	36	11.22	35.76	34	95.3	209.3	4.3	1
	Mexico	13,000	29	58	21.42	34.34	30	95.0	129.2	43.0	17
	Canada	13,000	32	74	25.74	25.11	15	95.4	36.6	34.3	43
**Europe**	UK	14,550	28	30	9.86	21.13	25	94.1	66.2	37.5	33
	France	15,294	29	70	26.25	29.53	25	94.1	65.0	30.5	28
	Germany	14,835	29	35	14.00	19.97	19	94.2	82.1	63.7	45
	Italy	14,000	29	19	8.29	43.53	19	94.6	59.4	36.8	34
	Spain	4,148	29	18	8.47	31.94	35	98.4	46.4	22.1	8
	Poland	6,672	29	60	25.26	33.28	17	97.4	38.2	17.2	12

Notations: PCs = private cars; Pop = population; M = millions;
D_{pc} = average annual driving distance per PC;
LT_{pc} = average annual time lost in traffic congestion per PC;
V_{pc} = estimated average road traffic speed of PCs;
%LT = percent of lost time in traffic congestion out of total commuting time;
%F = percentage of road accident fatalities out of all deaths by accidents;
%PM2.5 = percent of PM2.5 from road traffic out of total produced;
%P = percentage of time cars park;
ALoM = nonstop (24/7) active PCs per 1000 inhabitants.

Note: Complete coverage of the data elements including sources appear in Supplementary Table 8.

Supplementary Table 2. Characteristics and data, part 1, of areas presented by Figs. 2 and 3

**City** (by pop. density)	**Total city pop.** (people)	**Country**	**Examined zone**	**Zone's pop. density** (people/km^2)	**Selected zone's center location as starting point**
Mumbai	12,442,373	India	Santa Cruz West	27,223	Santa Cruz
Paris	2,240,000	France	Le Marais	21,000	Le Centre Pompidou
Seoul	9,774,000	S. Korea	City centre	17,000	Dongdaemum
Tokyo	9,273,000	Japan	Bunkyo	14,790	Tokyo Train Station
Mexico City	8,875,000	Mexico	Doctores	14,616	General Hospital
New York	8,538,000	USA	Lower Manhattan	10,858	W41st St.& 6th Ave.
Santiago	5.561,000	Chile	City center	9,339	Mall Plaza Alameda
Singapore	5,607,000	Singapore	City center	9,302	Nex Mall
Bangkok	5,782,000	Thailand	City center	7,561	State Tower
London	8,853,000	United Kingdom	City center	5,632	Queen Theatre
Chicago	2,695,598	American	CBD area	4,594	Randolph/Wabash
Toronto	2,731,571	Canada	Old Toronto	4,334	CF Toronto Eaton Centre
Shanghai	24,183,300	China	Jingan	6,000	Shanghai People's Square
Auckland	1,534,700	New Zealand	Auckland CBD	1,400	Britomart
Copenhagen	1,295,686	Denmark	Indre By	1,360	Norreport Station
Beijing	20,794,000	China	Guomao	1,293	Beijing Yintai Centre
Sydney	5,029,768	Australia	CBD area	400	Westfield Sydney

Supplementary Table 3. Characteristics and data, part 2, of areas presented by Figs. 2 and 3

**City** (by pop. density)	**Characteristics of the selected center**	**Land use characteristics**	**Ave. vehicle speed** (km/hr) **5-6PM**	**Year of data**
Mumbai	Train station center with an airport nearby; high PT demand	Train station, airports, shopping mall	19	2015
Paris	A busy center of traffic and the metro system	Shopping mall, tourism	22	2012
Seoul	Subway transit center with heavy traffic conditions	University and shopping center	19	2016
Tokyo	Center with a high PT demand	University, library, shopping mall offices	22	2016
Mexico City	A point with high PT and traffic demand	Commercial center, shopping mall, hospital	17	2013
New York	A busy central traffic area	University, library, shopping mall offices	20	2017
Santiago	A busy central area with a high demand for subway and bus services	Shopping mall, offices, government buildings	37	2014
Singapore	Central, high demand, point of subway and bus lines	Shopping mall, schools, tourism	24	2016
Bangkok	High PT demand point with a subway station nearby	Tourism, shopping mall, offices	24	2015
London	Central busy traffic and subway point	Theater, shopping mall, tourism	24	2017
Chicago	Center and major PT demand point	Parks, shopping malls, offices	34	2017
Toronto	High PT demand point with many rail stops nearby	University, library, shopping mall, tourism	35	2016
Shanghai	Central point of high PT and traffic demand with huge shopping malls	Shopping mall, offices	16	2017
Auckland	A busy rail and bus activities and demand point	Shopping mall, offices	27	2017
Copenhagen	Center and major PT station	Parks, shopping malls, offices	24	2012
Beijing	A busy traffic, subway and bus demands' point	Conference hall, shopping mall, offices	16	2017
Sydney	Central demand point of trains and buses	Shopping mall, offices	29	2017

Supplementary Table 4. Results of contour areas for four travel times used in Figs. 2 and 3

City no.	City (by pop. density)	**Total contour area** (km²) **for four T** (minutes)				**TBEC contour area** (km²) **for four T** (minutes)				**TBC contour area** (km²) **for four T** (minutes)				**CBT contour area** (km²) **for four T** (minutes)			
		**30**	**45**	**60**	**90**	**30**	**45**	**60**	**90**	**30**	**45**	**60**	**90**	**30**	**45**	**60**	**90**
1	Mumbai	53	62	64	103	42	40	48	62	27	20	15	7	11	22	16	41
2	Paris	36	103	189	251	30	86	162	207	7	31	77	71	6	17	27	44
3	Seoul	69	93	146	195	56	67	103	109	33	30	43	32	13	26	43	86
4	Tokyo	134	129	219	315	129	120	143	258	90	16	52	80	5	9	76	57
5	Mexico City	46	171	170	279	37	113	121	238	21	85	63	79	9	58	49	41
6	Shanghai	163	299	405	474	30	226	306	343	7	87	118	71	133	73	99	131
7	Beijing	26	56	109	153	20	54	88	110	8	23	31	14	6	2	21	43
8	New York	36	90	376	201	36	90	354	139	24	66	285	66	0	0	22	62
9	Santiago	61	126	129	205	44	75	74	134	18	45	14	14	17	51	55	71
10	Singapore	86	288	335	396	61	260	231	229	17	77	65	43	25	28	104	167
11	Bangkok	37	85	164	157	29	67	112	79	7	12	43	1	8	18	52	78
12	London	66	311	364	189	66	307	357	152	45	263	297	82	0	4	7	37
13	Chicago	127	73	103	131	124	45	57	63	103	8	7	6	3	28	46	68
14	Toronto	79	128	171	207	51	82	121	104	23	29	40	11	28	46	50	103
15	Sydney	52	158	285	300	32	88	137	117	9	23	24	9	20	70	148	183
16	Auckland	72	121	502	268	66	85	293	224	44	13	211	13	6	36	209	44
17	Copen-hagen	121	143	190	306	107	81	99	142	70	13	11	0	14	62	91	164

Notations: T=30, 45, 60, 90 minutes representing maximum travel time to the contour line of one of the four contour maps, where travel time is based on the shortest path routing from the city's center point.

CBT - cars are better (faster) than transit.

TBC - transit is better (faster) than cars.

TBEC - transit is better (faster) or equal to cars.

Supplementary Table 5. Characteristics of A, A1, and B of the 17-city

City (by pop. density)	Name of A	Name/ location of A1	Name/ location of B	A1 to B distance (km)
Mumbai	Andheri East	MIDC Police Station	Santa Cruz	7.6
Paris	Boulogne-Billancourt	Mairie	Le Centre Pompidou	10.1
Seoul	Seocho-gu	Nambu Bus Terminal	Dongdaemum Market	11.2
Tokyo	Edogawa City	Matsue Park	Tokyo Train Station	12.3
Mexico City	Naucalpan	Acueducto de los Remedios	General Hospital	14.6
Shanghai	Putuo	Back Qinjia Corner	Shanghai People's Square	13.4
Beijing	Wangjing	Futong Train Station	Beijing Yintai Centre	12.0
New York	Maspeth	St. Stanislaus Kostka School	Midtown Soho	9.0
Santiago	Las Condes	Pueblito Los Dominicos	Mall Plaza Alameda	18.5
Singapore	Sengkang	Springdale Primary School	Nex Mall	8.0
Bangkok	Wang Thonglang	Udomsuksa School	State Tower	17.0
London	Camberwell	Camberwell Library	Queen Theatre	7.8
Chicago	Humboldt Park	Harding (Frederick) Park	Randolph/Wabash	12.0
Toronto	York	Eglinton - Gilbert Parkette	CF Toronto Eaton Centre	10.5
Sydney	Marrickville	Marrickville Town Hall	Westfield Sydney	9.5
Auckland	Mount Roskill	Dominion Road School	Britomart	11.0
Copenhagen	Vanløse	Børglumvej (Jernbane Allé)	Norreport Station	8.2

Supplementary Table 6. Number of automated vehicles out of 1000 PCs for three cases represented in Fig. 5

City (by pop. density)	[%APTV, %IAPTV]														
	[100,0]					[50,50]					[0,100]				
	APTVs *a*	IAV-PTs *b*	IAPTVs *c*	Total *d*= *a*+*c*	Total in % *e*	*a*	*b*	*c*	*d*	*e*	*a*	*b*	*c*	*d*	*e*
Mumbai	32	62	0	32	3%	17	52	350	367	37%	0	0	700	700	70%
Paris	31	91	0	31	3%	16	45	317	333	33%	0	0	633	633	63%
Seoul	28	45	0	28	3%	19	35	263	282	28%	0	0	525	525	53%
Tokyo	29	62	0	29	3%	15	36	267	282	28%	0	0	533	533	53%
Mexico City	33	83	0	33	3%	17	42	363	380	38%	0	0	725	725	73%
Shanghai	32	62	0	32	3%	17	41	333	350	35%	0	0	667	667	67%
Beijing	31	70	0	31	3%	16	36	300	316	32%	0	0	630	630	63%
New York	32	62	0	32	3%	17	33	313	330	33%	0	0	625	625	63%
Santiago	31	77	0	31	3%	16	55	308	324	32%	0	0	617	617	62%
Singapore	31	87	0	31	3%	15	43	292	307	31%	0	0	583	583	58%
Bangkok	31	95	0	31	3%	15	48	296	311	31%	0	0	592	592	59%
London	31	100	0	31	3%	15	50	317	332	33%	0	0	633	633	63%
Chicago	29	80	0	29	3%	14	42	250	264	26%	0	0	500	500	50%
Toronto	30	111	0	30	3%	14	56	254	268	27%	0	0	508	508	51%
Sydney	27	62	0	27	3%	12	31	221	233	23%	0	0	442	442	44%
Auckland	22	50	0	22	2%	11	29	229	240	24%	0	0	458	458	46%
Copenhagen	30	125	0	30	3%	14	63	267	281	28%	0	0	533	533	53%

Notations: APTV - automated PT vehicle, i.e., shared automated bus.
IAPTV - individual automated PT vehicle, i.e., automated taxi-type company car.
IAV-PT - individual automated vehicle of local PT service, i.e., automated local company car to feed into, or distribute from, an APTV.

Supplementary Table 7. Number of automated vehicles out of 1000 PCs for two additional cases

City (by pop. density)	[%APTV, %IAPTV]									
	[75,25]					[25,75]				
	APTVs a	IAV-PTs *b*	IAPTVs *c*	Total *d*= *a*+*c*	Total in % *e*	*a*	*b*	*c*	*d*	*e*
Mumbai	22	94	133	155	16%	8	31	400	408	41%
Paris	22	83	127	149	15%	7	28	381	388	39%
Seoul	20	35	131	151	15%	19	35	394	413	41%
Tokyo	25	52	175	200	20%	11	52	525	536	54%
Mexico City	23	71	148	171	17%	11	24	444	455	46%
Shanghai	24	47	156	180	18%	16	33	469	485	49%
Beijing	23	75	158	181	18%	8	48	75	83	8%
New York	25	63	181	206	21%	11	41	544	555	56%
Santiago	22	63	125	147	15%	7	23	375	382	38%
Singapore	24	47	167	191	19%	16	41	500	516	52%
Bangkok	23	58	154	177	18%	8	55	463	471	47%
London	24	68	158	182	18%	15	26	475	490	49%
Chicago	23	53	156	179	18%	8	27	469	477	48%
Toronto	23	65	146	169	17%	10	32	438	448	45%
Sydney	21	47	133	154	15%	15	36	400	415	42%
Auckland	20	47	110	130	13%	9	22	331	340	34%
Copenhagen	17	40	115	132	13%	6	23	344	350	35%

Supplementary Table 8. Information for **Supplementary Table 1 with list of references/sources indicated by superscripts**

Group	Country	D_{pc} (km)	V_{pc} (km/h)	LT_{pc} (hr/year)	%LT	%F	%PM2.5	%P	Pop (M)	# PCs (M)	ALoM
**Asia**	China	15,000[2] (2015)*	26[58] (2016)	80[20, 21] (2017)	33.68%	61.49%[37] (2016)	17%[56] (2014)	93.5%	1,409.5[57] (2017)	217.0[59] (2018)	10
	Japan	18,000[3] (2014)	24[58] (2016)	78[19] (2017)	48.00%	13.99%[38] (2016)	23%[56] (2014)	91.3%	127.5[57] (2017)	78.1[60] (2017)	54
	Singapore	16,700[4] (2016)	29[58] (2016)	87[22] (2015)	23.73%	33.77%[39] (2016)	36%[56] (2014)	93.5%	5.7[57] (2017)	1.0[61] (2017)	12
	India	20,000[5] (2016)	35[58] (2016)	60[23] (2017)	15.82%	42.82%[40] (2016)	37%[56] (2014)	93.5%	1,339.2[57] (2017)	191.0[62] (2016)	9
	Korea	13,651[6] (2015)	32[58] (2016)	107[24] (2017)	34.70%	57.81%[41] (2016)	23%[56] (2014)	95.2%	51.0[57] (2017)	22.8[63] (2018)	22
**Oceania**	Australia	15,530[7] (2013)	32[58] (2016)	94[25] (2013)	28.20%	32.33%[42] (2016)	26%[56] (2014)	94.5%	24.5[57] (2017)	19.2[64] (2018)	44
	New Zealand	10,400[16] (2014)	24[58] (2016)	80[26] (2018)	32.54%	28.57%[43] (2016)	26%[56] (2014)	95.0%	4.7[57] (2017)	3.8[65] (2015)	50
**Africa**	South Africa	20,000[8] (2017)	35[58] (2016)	90[27] (2014)	25.71%	38.84%[44] (2016)	17%[56] (2014)	93.5%	56.7[57] (2017)	12.0[66] (2017)	14
	Egypt	14,000[9] (2014)	35[58] (2016)	76[28] (2015)	21.71%	54.35%[45] (2016)	17%[56] (2014)	95.5%	97.6[57] (2017)	9.4[67] (2017)	4
**America**	USA	18,000[10] (2015)	47[58] (2016)	42[17] (2015)	12.76%	38.30%[46] (2016)	24%[56] (2014)	95.6%	324.5[57] (2017)	268.8[68] (2017)	36
	Brazil	12,000[11] (2013)	29[58] (2016)	36[29] (2018)	11.22%	35.76%[47] (2016)	34%[56] (2014)	95.3%	209.3[57] (2017)	4.3[69] (2018)	1
	Mexico	13,000[12] (2015)	29[58] (2016)	58[30] (2018)	21.42%	34.34%[48] (2016)	30%[56] (2014)	95.0%	129.2[57] (2017)	43.0[70] (2018)	17
	Canada	13,000[13] (2015)	32[58] (2016)	74[31] (2010)	25.74%	25.11%[49] (2016)	15%[56] (2014)	95.4%	36.6[57] (2017)	34.3[71] (2017)	43
**Europe**	UK	14,550[13] (2015)	28[58] (2016)	30[18] (2015)	9.86%	21.13%[50] (2016)	25%[56] (2014)	94.1%	66.2[57] (2017)	37.5[72] (2017)	33
	France	15,294[13] (2015)	29[58] (2016)	70[32] (2018)	26.25%	29.53%[51] (2016)	25%[56] (2014)	94.1%	65.0[57] (2017)	30.5[73] (2017)	28
	Germany	14,835[13] (2016)	29[58] (2016)	35[33] (2018)	14.00%	19.97%[52] (2016)	19%[56] (2014)	94.2%	82.1[57] (2017)	63.7[74] (2018)	45
	Italy	14,000[14] (2014)	29[58] (2016)	19[34] (2016)	8.29%	43.53%[53] (2016)	19%[56] (2014)	94.6%	59.4[57] (2017)	36.8[75] (2016)	34

	Spain	4,148[13] (2015)	29[58] (2016)	18[35] (2016)	8.47%	31.94%[54] (2016)	35%[56] (2014)	98.4%	46.4[57] (2017)	22.1[76] (2015)	8
	Poland	6,672[15] (2015)	29[58] (2016)	60[36] (2015)	25.26%	33.28%[55] (2016)	17%[56] (2014)	97.4%	38.2[57] (2017)	17.2[77] (2017)	12

Notations: PCs = private cars; Pop = population; M = millions; D_{pc} = average annual driving distance per a PC;
LT_{pc} = average annual time lost in traffic congestion per a PC; V_{pc}= estimated average PC road traffic speed;
%LT = percent of total commuting lost time in traffic congestion;
%F = percentage of road accident fatalities out of all deaths by accidents;
%PM2.5 = percent of PM2.5 from road traffic out of total produced;
%P = percentage of time cars park;
ALoM = nonstop (24/7) active PCs per 1000 inhabitants.

*Year of data shown in each cell of the table; sources of data appear in the following list.

References/Sources

1. https://en.wikipedia.org/wiki/List_of_countries_by_vehicles_per_capita
2. http://www.wrirosscities.org/sites/default/files/China%20Motorization%20Trends%20-%20Chapter%204.pdf
3. https://www.statista.com/statistics/645317/japan-share-passenger-car-travel-distance/
4. https://www.straitstimes.com/lifestyle/motoring/drivers-no-longer-going-the-distance
5. https://www.quora.com/What-is-the-average-yearly-distance-driven-in-a-passenger-car-in-India
6. http://www.cvinfo.com/news/articleView.html?idxno=3044 (using Google translation, in Korean)
7. http://www.roymorgan.com/findings/australian-moterists-drive-average-15530km-201305090702
8. https://investorchallenge.co.za/what-is-the-average-south-africans-biggest-expense/
9. https://www.vda.de/dam/vda/publications/2015/automation.pdf
10. https://www.fool.com/investing/general/2015/01/25/the-average-american-drives-this-much-each-year-ho.aspx
11. onlinepubs.trb.org/Onlinepubs/trr/1983/898/898-057.pdf
12. https://www.ielts.org/-/media/pdfs/115013_academic_reading_sample_task_-_matching_information__2_.ashx?la=en
13. https://uk.nissannews.com/en-GB/releases/release-128282-nissan-s-electric-car-drivers-cover-more-miles-than-the-average-petrol-or-diesel-vehicle
14. https://www.statista.com/statistics/692853/average-annual-distance-covered-by-drivers-in-italy/
15. ec.europa.eu/environment/enveco/auto-oil/pdf/aopces_es.pdf
16. https://transport.govt.nz/assets/Uploads/Research/Documents/Drivers-2015.pdf
17. https://www.autoinsurancecenter.com/traffic-jammed.htm
18. https://www.fleetnews.co.uk/news/fleet-industry-news/2016/03/15/uk-drivers-spent-30-hours-in-congestion-in-2015
19. https://newsphere.jp/economy/20170427-4/ (using Google translation, in Japanese)

20. http://cn-hangzhou.oss-pub.aliyun-inc.com/download-report/download/yearly_report/2017/2017%E5%B9%B4%E4%B8%AD%E5%9B%BD%E4%B8%BB%E8%A6%81%E5%9F%8E%E5%B8%82%E4%BA%A4%E9%80%9A%E5%88%86%E6%9E%90%E6%8A%A5%E5%91%8A-final%E7%89%88.pdf (using Google translation, in Chinese)
21. http://www.chinanews.com/sh/2015/01-26/7005909.shtml (using Google translation, in Chinese)
22. https://mustsharenews.com/time-wasted-on-traffic-jams/
23. https://timesofindia.indiatimes.com/city/bengaluru/10-million-bengalureans-lose-60-crore-hours-rs-3700-crore-a-year-to-road-congestion/articleshow/56368901.cms
24. http://www.edaily.co.kr/news/news_detail.asp?newsId=02430486616057496&mediaCodeNo=257 (using Google translation, in Korean)
25. https://www.news.com.au/travel/travel-updates/australian-motorists-spend-94-hours-a-year-in-congestion-tomtom-traffic-index/news-story/30a81e41178911bffc00f1380383153f
26. https://www.radionz.co.nz/news/national/351558/commuters-waste-80-hours-stuck-in-traffic
27. https://www.cnbcafrica.com/news/southern-africa/2015/04/02/south-africa-traffic-congestion-tomtom-index/
28. https://www.systemdynamics.org/assets/conferences/2015/proceed/papers/P1312.pdf
29. https://www.cbsnews.com/pictures/worst-traffic-cities-in-the-world/23/
30. https://www.cbsnews.com/pictures/worst-traffic-cities-in-the-world/11/
31. http://www.wikiwand.com/en/Traffic_congestion
32. http://inrix.com/press-releases/2661/
33. http://inrix.com/press-releases/2663/
34. https://www.wantedinmilan.com/news/milan-italys-worst-for-traffic.html
35. https://www.autoescuelago.com/ciudades-atascos/ (using Google translation, in Spanish)
36. www.inrixtraffic.com/feed/
37. https://www.worldlifeexpectancy.com/country-health-profile/china
38. https://www.worldlifeexpectancy.com/country-health-profile/japan
39. https://www.worldlifeexpectancy.com/country-health-profile/singapore
40. https://www.worldlifeexpectancy.com/country-health-profile/india
41. https://www.worldlifeexpectancy.com/country-health-profile/korea
42. https://www.worldlifeexpectancy.com/country-health-profile/australia
43. https://www.worldlifeexpectancy.com/country-health-profile/new-zealand
44. https://www.worldlifeexpectancy.com/country-health-profile/south-africa
45. https://www.worldlifeexpectancy.com/country-health-profile/egypt
46. https://www.worldlifeexpectancy.com/country-health-profile/united-states
47. https://www.worldlifeexpectancy.com/country-health-profile/brazil
48. https://www.worldlifeexpectancy.com/country-health-profile/mexico
49. https://www.worldlifeexpectancy.com/country-health-profile/canada
50. https://www.worldlifeexpectancy.com/country-health-profile/united-kingdom
51. https://www.worldlifeexpectancy.com/country-health-profile/france
52. https://www.worldlifeexpectancy.com/country-health-profile/germany
53. https://www.worldlifeexpectancy.com/country-health-profile/italy
54. https://www.worldlifeexpectancy.com/country-health-profile/spain

55. https://www.worldlifeexpectancy.com/country-health-profile/poland
56. Karagulian, F., Belis, C. A., Dora, C. F. C., Prüss-Ustün, A. M., Bonjour, S., Adair-Rohani, H., & Amann, M. (2015). Contributions to cities' ambient particulate matter (PM): A systematic review of local source contributions at global level. Atmospheric environment, 120, 475-483.
57. https://en.wikipedia.org/wiki/List_of_countries_by_population_
58. http://chartsbin.com/view/41749
59. https://www.statista.com/statistics/285306/number-of-car-owners-in-china/
60. https://www.statista.com/statistics/674574/japan-motor-vehicle-in-use-numbers/
61. https://www.singstat.gov.sg/-/media/files/publications/households/pp-s24.pdf
62. https://www.statista.com/statistics/664729/total-number-of-vehicles-india/
63. https://www.ceicdata.com/en/korea/number-of-registered-motor-vehicles/registered-motor-vehicles
64. https://www.abs.gov.au/ausstats/abs@.nsf/mf/9309.0
65. http://www.ehinz.ac.nz/assets/Factsheets/Released-2017/EHI8-9-NumberOfVehiclesInNZ2000-2015-release-201701.pdf
66. https://www.wheels24.co.za/News/Industry_News/youll-never-guess-how-many-vehicles-are-registered-in-sa-20170328
67. http://english.ahram.org.eg/NewsContent/1/64/270451/Egypt/Politics-/-million-licensed-vehicles-in-Egypt-CAPMAS.aspx
68. https://www.census.gov/history/www/homepage_archive/2018/october_2018.html
69. https://www.volkswagen-newsroom.com/en/press-releases/volkswagen-group-deliveries-reach-all-time-high-392
70. https://oxfordre.com/latinamericanhistory/view/10.1093/acrefore/9780199366439.001.0001/acrefore-9780199366439-e-456
71. https://www150.statcan.gc.ca/n1/daily-quotidien/180615/dq180615e-eng.htm
72. https://www.gov.uk/government/statistics/vehicle-licensing-statistics-january-to-march-2017
73. https://www.statista.com/statistics/455887/passenger-cars-registered-in-france/
74. https://www.best-selling-cars.com/germany/2018-germany-total-number-registered-cars/
75. https://knoema.com/atlas/ranks/Private-car-theft-rate
76. https://www.statista.com/statistics/452310/spain-number-of-registered-passenger-cars/
77. https://www.acea.be/uploads/statistic_documents/ACEA_Report_Vehicles_in_use-Europe_2017.pdf

Supplementary Table 9. **Information on data sources and input data for ArcGIS 10.3.1**

Notes:

(a) The speeds of all main/major/general roads are average speeds as they appear in the sources.
(b) The speeds appearing for the main/minor/general roads are those found for the majority (or all) of the roads.
(c) Sources could not be found for some of the minor roads, and thus, their design speed was assumed.
(d) General speed is for a general category road between main and minor roads.
(e) Restraining and congestion refer to data on traffic congestion and/or traffic restrictions.

**City**	**City name** (by pop. density)			
**1**	**Mumbai**	Map	OpenStreetMap	http://www.openstreetmap.org.nz/
			Google Map	maps.google.com
			TomTom City	https://www.tomtom.com/en_gb/traffic-news/
			World Map	http://www.arcgis.com/home/item.html?id=94f838a535334cf1aa061846514b77c7
		Main road speed	17 km/hr	https://www.tomtom.com/en_gb/traffic-news/mumbai-traffic/traffic-flow
		Minor road speed	20 km/hr	https://www.tomtom.com/en_gb/traffic-news/mumbai-traffic/traffic-flow
		General speed	25 km/hr	https://www.tomtom.com/en_gb/traffic-news/mumbai-traffic/traffic-flow
		Restraining & congestion		https://timesofindia.indiatimes.com/topic/Traffic-congestion-in-Mumbai<br>https://timesofindia.indiatimes.com/city/delhi/congestion-fee-to-unclog-some-roads/articleshow/61886317.cms -
		All PT timetables		
		Other		https://en.wikipedia.org/wiki/Public_transport_in_Mumbai
**2**	**Paris**	Map	OpenStreetMap	http://www.openstreetmap.org.nz/
			Google Map	maps.google.com
			TomTom City	https://www.tomtom.com/en_gb/traffic-news/
			World Map	http://www.arcgis.com/home/item.html?id=94f838a535334cf1aa061846514b77c7
		Main road speed	23 km/hr	https://www.tomtom.com/en_gb/traffic-news/paris-traffic/traffic-flow<br>http://www.parisaeroport.fr/docs/default-source/groupe-fichiers/presse/cp-juillet-septembre-2017/pr-12092017-traffic-figures-august-2017.pdf?sfvrsn=2<br>https://www.franceix.net/en/technical/traffic-statistics/
		Minor road speed	30 km/hr	https://www.tomtom.com/en_gb/traffic-news/paris-traffic/traffic-flow
		General speed	27 km/hr	https://www.tomtom.com/en_gb/traffic-news/paris-traffic/traffic-flow<br>http://www.parisaeroport.fr/en/group/finance/investor-relations/traffic
		Restraining & congestion		https://www.tomtom.com/en_gb/traffic-news/paris-traffic/traffic-flow<br>http://ec.europa.eu/eurostat/statistics-

		All PT timetables		
		Other		http://www.asianhumannet.org/db/datas/10_transport/planning_en.pdf
**3**	**Seoul**	Map	OpenStreetMap	http://www.openstreetmap.org.nz/
			Google Map	maps.google.com
			TomTom City	https://www.tomtom.com/en_gb/traffic-news/
			World Map	http://www.arcgis.com/home/item.html?id=94f838a535334cf1aa061846514b77c7
		Main road speed	15 km/hr	https://english.koti.re.kr/
		Minor road speed	23 km/hr	https://english.koti.re.kr/
		General speed	28 km/hr	https://github.com/SeoulTech/open-data-seoul#transportation
				https://www.data.go.kr/search/index.do
		Restraining & congestion		http://www.unikorea.go.kr/eng_unikorea/relations/statistics/traffic/
		All PT timetables		http://topis.seoul.go.kr/eng/english.jsp
		Other		http://english.seoul.go.kr/policy-information/traffic/major-traffic-statistics/
**4**	**Tokyo**	Map	OpenStreetMap	http://www.openstreetmap.org.nz/
			Google Map	maps.google.com
			TomTom City	https://www.tomtom.com/en_gb/traffic-news/
			World Map	http://www.arcgis.com/home/item.html?id=94f838a535334cf1aa061846514b77c7
		Main road speed	18.5 km/h	http://journal.jp.fujitsu.com/en/2015/08/27/01/
		Minor road speed	22 km/hr	http://www.trafficindex.org/tokyo/
		General speed	30 km/hr	
				http://nlftp.mlit.go.jp/ksj-e/gml/datalist/KsjTmplt-S05-c.html
				https://www.distancecalculator.net/from-osaka-shi-to-tokyo
		Restraining & congestion		http://nlftp.mlit.go.jp/ksj-e/gml/cgi-bin/download.php<br>http://nlftp.mlit.go.jp/ksj-e/gml/datalist/KsjTmplt-S05-c.html<br>http://trafficdata.iis.u-tokyo.ac.jp/repository.php?loc=66
				http://www.mlit.go.jp/road/management-e/e_pdf/0403_3.pdf
		All PT timetables		
		Other		http://www.asianhumannet.org/db/datas/10_transport/planning_en.pdf
**5**	**Mexico City**	Map	OpenStreetMap	http://www.openstreetmap.org.nz/
			Google Map	maps.google.com
			TomTom City	https://www.tomtom.com/en_gb/traffic-news/
			World Map	http://www.arcgis.com/home/item.html?id=94f838a535334cf1aa061846514b77c7

	Main road speed	17 km/hr	https://www.tomtom.com/en_gb/traffic-news/mexico-city-traffic/traffic-flow
	Minor road speed	20 km/hr	https://www.tomtom.com/en_gb/traffic-news/mexico-city-traffic/traffic-flow
	General speed	23 km/hr	https://www.tomtom.com/en_gb/traffic-news/mexico-city-traffic/traffic-flow
	Restraining & congestion		https://www.tomtom.com/en_gb/traffic-news/mexico-city-traffic/traffic-flow<br>https://www.transitwiki.org/TransitWiki/index.php/Publicly
	Other		http://www.accessmagazine.org/articles/spring-2015/suburban-transit-in-mexico-city/<br>http://www.accessmagazine.org/articles/spring-2015/suburban-transit-in-mexico-city/
**6 Shanghai**	Map	OpeStreetMap	http://www.openstreetmap.org.nz/
		Google Map	maps.google.com
		Baidu Map	https://maps.baidu.com/
		Part of the map	http://download.csdn.net/download/u012222998/6309629
		Gaode Map	https://ditu.amap.com/
		World Map	http://www.arcgis.com/home/item.html?id=94f838a535334cf1aa061846514b77c7
	Main road speed	13-17 km/hr	http://www.jtcx.sh.cn/trafficanalyse.html
	General speed	15 km/hr	https://wenku.baidu.com/view/d591ccd06037ee06eff9aef8941ea76e58fa4ae5.html<br>http://www.sohu.com/a/131413091_182825<br>http://www.jfdaily.com/news/detail?id=50054
	Restraining & congestion		http://sh.sina.com.cn/news/m/2018-01-19/detail-ifyqtwzu6809008-p5.shtml?from=wap<br>http://report.amap.com/predict/holiday/predict.do#
	All PT timetables		A few stops chosen to calculate average bus travel time.<br>http://bus.mapbar.com/shanghai/xianlu/<br>Subway system travel speed, 60 km/hr<br>https://wenku.baidu.com/view/6762c56c87c24028905fc338.html
**7 Beijing**	Map	OpenStreetMap	http://www.openstreetmap.org.nz/
		Google Map	maps.google.com
		Baidu Map	https://maps.baidu.com/
		Map of Beijing	http://www.pudn.com/Download/item/id/1722941.html
		World Map	http://www.arcgis.com/home/item.html?id=94f838a535334cf1aa061846514b77c7
	Average travel speed	15 km/hr	https://www.tomtom.com/en_gb/traffic-index/beijing-traffic
	Restraining & congestion		http://www.bjdata.gov.cn/cms/web/download/NohistoryDownload.jsp?articleID=4677<br>http://www.bjdata.gov.cn/cms/web/bjdata/loginDoSomething/dengluLogin.jsp

		PT-Subway		Use the timetables to get the average travel speed between stations. Average wait time is 2 minutes.<br>http://map.sogou.com/index.html#c=12940924,4859575,11&lq=%u5730%u94C1%u660C%u5E73%u7EBF&where=12913250,4840656.25,12974750,4873093.75,0&page=1,10<br>Train travel at 80 km/hr.
		Bus timetables		A few stops selected to calculate average bus travel time.<br>http://bus.mapbar.com/beijing/xianlu/
		Other		http://www.baidu.com/link?url=JHYvgK_bulnvt6EPIjx3AlZY34faVlbnXDQT-PdHDYIkyhX6iYT_18Kj1psxVOfSI99VcQPjwgXE8W9yeAF_rxeFjoiCdCnPJA5mY4WUHeG&wd=&eqid=fb29479c0070e3de000000045891aea7
**8**	**New York City**	Map	OpenStreetMap	http://www.openstreetmap.org.nz/
			Google Map	maps.google.com
			Government data	https://opendata.cityofnewyork.us/<br>https://data.cityofnewyork.us/City-Government/Georeferenced-NYC-Zoning-Maps/mxbm-493w
			PT Map	https://data.cityofnewyork.us/Transportation/Subway-Stations/arq3-7z49
			World Map	http://www.arcgis.com/home/item.html?id=94f838a535334cf1aa061846514b77c7
		Average travel speed	8-12 km/hr	https://data.cityofnewyork.us/Transportation/Real-Time-Traffic-Speed-Data/qkm5-nuaq
		Restraining & congestion	Government data	https://data.cityofnewyork.us/Transportation/Real-Time-Traffic-Speed-Data/qkm5-nuaq
			Real time data	http://dotsignals.org/
		PT-Subway	Real time data	http://dotsignals.org/
		Bus timetables	PT data	https://data.cityofnewyork.us/Transportation/Subway-Stations/arq3-7z49
		Other		http://www.chinadaily.com.cn/china/2012cpc/2012-11/10/content_15910395.htm
**9**	**Santiago**	Map	OpenStreetMap	http://www.openstreetmap.org.nz/
			Google Map	maps.google.com
			TomTom City	https://www.tomtom.com/en_gb/traffic-news/
			World Map	http://www.arcgis.com/home/item.html?id=94f838a535334cf1aa061846514b77c7<br>https://www.viamichelin.com/web/Traffic/Traffic_info-Santiago-_-Region_Metropolitana_de_Santiago-Chile
		Main road speed	21 km/hr	https://www.caminoadventures.com/camino-de-santiago-statistics/

				https://www.waze.com/driverindex
		Minor road speed	27 km/hr	
		General speed	24 km/hr	https://www.caminoadventures.com/camino-de-santiago-statistics/
		Restraining & congestion		
		Other		http://www.worldbank.org/en/results/2013/04/11/Urban-Transport-in-Santiago
				http://unhabitat.org/wp-content/uploads/2013/06/GRHS.2013.Case_.Study_.Santiago.Chile_.pdf
**10**	**Singapore**	Map	OpenStreetMap	http://www.openstreetmap.org.nz/
			Google Map	maps.google.com
			TomTom City	https://www.tomtom.com/en_gb/traffic-news/
			World Map	http://www.arcgis.com/home/item.html?id=94f838a535334cf1aa061846514b77c7
		Main road speed	25 km/hr	https://www.tomtom.com/en_gb/trafficindex/city/singapore
				https://www.singstat.gov.sg/statistics/latest-data#9
		Minor road speed	27 km/hr	https://www.tomtom.com/en_gb/trafficindex/city/singapore
		General speed	25 km/hr	
				https://www.lta.gov.sg/content/ltaweb/en/roads-and-motoring.html
		Restraining & congestion		
		All PT timetables		https://www.sbstransit.com.sg/transport/trpt_bus_timetable.aspx
				https://www.mytransport.sg/content/mytransport/home/dataMall.html
				https://www.lta.gov.sg/content/ltaweb/en/public-transport/mrt-and-lrt-trains/train-system-map.html
**11**	**Bangkok**	Map	OpenStreetMap	http://www.openstreetmap.org.nz/
			Google map	maps.google.com
			TomTom City	https://www.tomtom.com/en_gb/traffic-news/
			World Map	http://www.arcgis.com/home/item.html?id=94f838a535334cf1aa061846514b77c7
		Main road speed	24 km/hr	https://www.ontario.ca/data/2011-average-car-travel-speed
		Minor road speed	26 km/hr	
		General speed	27 km/hr	
				https://coconuts.co/jakarta/news/average-driving-speed-jakarta-weekday-mornings-5-kmh/

		Restraining & congestion		http://inrix.com/scorecard-city/?city=Bangkok&index=16
				http://www.straitstimes.com/asia/se-asia/thailand-has-worlds-most-congested-roads-survey
		All PT timetables		
				http://unhabitat.org/wp-content/uploads/2013/06/GRHS.2013.Case_.Study_.Bangkok.Thailand.pdf
		Other		
**12**	**London**	Map	OpenStreetMap	http://www.openstreetmap.org.nz/
			Google Map	maps.google.com
			Government data	https://tfl.gov.uk/
				https://data.gov.uk/dataset/gb-road-traffic-counts
				https://data.gov.uk/dataset/gb-road-traffic-counts/resource/e85266b0-4c7b-4f5b-b2db-22c57f9b952c
			World Map	http://www.arcgis.com/home/item.html?id=94f838a535334cf1aa061846514b77c7
		Main road speed	31 km/hr	http://www.dft.gov.uk/traffic-counts/download.php
		Minor road speed	15 km/hr	http://www.dft.gov.uk/traffic-counts/download.php
		General speed	32 km/hr	https://data.gov.uk/dataset/gb-road-traffic-counts
				https://www.gov.uk/government/collections/road-traffic-statistics#publications-2017
		Restraining & congestion		https://londontransportdata.wordpress.com/2012/06/25/traffic-speeds-and-congestion-by-region/
				https://data.london.gov.uk/dataset/traffic-flows-borough
				https://data.london.gov.uk/dataset/cycle-flows-tfl-road-network
		PT travel data		
				https://tfl.gov.uk/info-for/open-data-users/our-open-data#on-this-page-3
				https://data.london.gov.uk/dataset/public-transport-journeys-type-transport
				https://data.london.gov.uk/dataset/traffic-flows-borough
		Other		http://content.tfl.gov.uk/Travel-in-London-report-1.pdf
				http://search.abs.gov.au/s/search.html?clicked_fluster=public+transport+use&cluster0=public+transport&form=simple&origin=-
**13**	**Chicago**	Map	OpenStreetMap	http://www.openstreetmap.org.nz/
			Google Map	maps.google.com
			TomTom City	https://www.tomtom.com/en_gb/traffic-news/

			World Map	http://www.arcgis.com/home/item.html?id=94f838a535334cf1aa061846514b77c7
				http://gis.dot.illinois.gov/StateMapViewer/
		Main road speed	33 km/hr	https://www.tomtom.com/en_gb/trafficindex/city/chicago
		Minor road speed	40 km/hr	https://www.tomtom.com/en_gb/trafficindex/city/chicago
		General speed	37 km/hr	https://www.tomtom.com/en_gb/trafficindex/city/chicago
				https://www.travelmidwest.com/lmiga/map.jsp?mapname=chicagoArea
				http://www.cmap.illinois.gov/data/transportation/traffic/historic-traffic-data
		Restraining & congestion		http://udforu.com/in-progress/chicago-traffic-congestion-study/
				https://data.cityofchicago.org/Transportation/Average-Daily-Traffic-Counts-Map/pf56-35rv/data
				https://catalog.data.gov/dataset?tags=traffic&publisher=data.cityofchicago.org&organization=city-of-chicago
		All PT timetables		https://www.transitchicago.com/schedules/
**14**	**Toronto**	Map	OpenStreetMap	http://www.openstreetmap.org.nz/
			Google Map	maps.google.com
			TomTom City	https://www.tomtom.com/en_gb/traffic-news/
			World Map	http://www.arcgis.com/home/item.html?id=94f838a535334cf1aa061846514b77c7
		Main road speed	35km/hr	https://www.toronto.ca/wp-content/uploads/2017/11/91f0-city_congestion_trends_09-15-2015.pdf
				http://www.civil.uwaterloo.ca/bhellinga/publications/Publications/ITE-1994-Speed-delay.pdf
		Minor road speed	40km/hr	https://www.tomtom.com/en_gb/trafficindex/city/toronto
		General speed	39km/hr	https://www.tomtom.com/en_gb/trafficindex/city/toronto
				http://www.cbc.ca/news/technology/three-in-4-drivers-travel-above-the-speed-limit-on-canada-s-busiest-highway-1.3280254
		Restraining & congestion		https://www.thestar.com/news/gta/2015/10/09/congestion-in-toronto-still-a-headache-report.html
				https://torontoist.com/2016/09/the-real-cost-of-congestion-in-toronto/
				https://www.theglobeandmail.com/news/toronto/constant-gridlock/article18406658/
				http://www.metrolinx.com/en/regionalplanning/costsofcongestion/costs_congestion.aspx
		All PT timetables		https://ttc.ca/Routes/index.jsp

**15**	**Sydney**	Map	OpenStreetMap	http://www.openstreetmap.org.nz/
			Google Map	maps.google.com
				http://www.rms.nsw.gov.au/about/corporate-publications/statistics/traffic-volumes/aadt-map/index.html#/?z=7&lat=-33.78977232045953&lon=151.3446739999999&st=2
			PT stations	
				http://roadsreport.rms.nsw.gov.au/#/chart?location=NSW&dateStart=2017-09-01&dateEnd=2017-11-30&peak=AM&type=period-plot
			World Map	http://www.arcgis.com/home/item.html?id=94f838a535334cf1aa061846514b77c7
				http://www.rms.nsw.gov.au/documents/about/corporatepublications/statistics/annual-speed-traffic-volume-data-2009-2010.pdf
		Average travel speed	31 km/hr	
				http://roadsreport.rms.nsw.gov.au/#/chart?location=NSW&dateStart=2017-09-01&dateEnd=2017-11-30&peak=AM&type=period-plot
		Average Highway speed	40.5 km/hr	http://roadsreport.rms.nsw.gov.au/#/chart?location=NSW&dateStart=2017-12-01&dateEnd=2018-02-28&peak=AM&type=period-plot
		PT travel data		https://data.nsw.gov.au/data/dataset/train-data
				https://www.transport.nsw.gov.au/
				https://www.transport.nsw.gov.au/data-and-research
				http://search.abs.gov.au/s/search.html?clicked_fluster=public+transport+use&cluster0=public+transport&form=simple&origin=-9.483307%2C176.91669&profile=_default&query=%60Public+transport+use%60&collection=abs
		Other		
**16**	**Auckland**	Map	OpenStreetMap	http://www.openstreetmap.org.nz/
				https://wiki.openstreetmap.org/wiki/WikiProject_New_Zealand#OSM_Auckland_meetings
			Road maps/levels	
			Google Map	https://www.google.co.nz/maps?source=tldsi&hl=zh-CN
			Changes & revise	Use Map-editor to change data from internet; to suit the requirements.
			World Map	http://www.arcgis.com/home/item.html?id=94f838a535334cf1aa061846514b77c7
		Main road speed	42 km/hr	https://at.govt.nz/media/970611/Penlink-Traffic-and-Economic-Analysis.pdf
		Minor road speed	30 km/hr	
		General speed	Speed limits	Restrictions are not assigned to some road layers.
				https://at.govt.nz/media/1973943/schedule-of-speed-limits-27-june-2012.pdf
				Graphs of state highway MADT percentage changes (all vehicles) 2013.

			https://www.nzta.govt.nz/assets/resources/state-highway-traffic-volumes/docs/2013-2017-national-telemetry-site-profiles.pdf
			http://www.transport.govt.nz/research/roadsafetysurveys/speedsurveys/2013speedsurveyresultscarspeeds/
	Restraining & congestion		
			https://www.nzta.govt.nz/assets/resources/state-highway-traffic-volumes/docs/2013-2017-national-telemetry-site-profiles.pdf
			http://www.transport.govt.nz/ourwork/tmif/transport-volume/
			https://www.greaterauckland.org.nz/our-analysis/transport-statistics/traffic-volumes/
	All PT timetables		https://at.govt.nz/bus-train-ferry/timetables/#Central
	Other		http://transportblog.co.nz/tag/patronage/
**17 Copenhagen**	Map	OpenStreetMap	http://www.openstreetmap.org.nz/
			https://www.openstreetmap.org/node/13707878#map=11/55.6414/12.5910&layers=TND
		Road maps/levels	
			https://www.google.com/maps/place/Copenhagen,+Denmark/@55.6802826,12.5254926,12.25z/data=!4m5!3m4!1s0x4652533c5c803d23:0x4dd7edde69467b8!8m2!3d55.6760968!4d12.5683372?hl=en
		Google Map	
		Changes & revise	Use Map Editor to change data from Internet to suit requirements.
		World Map	http://www.arcgis.com/home/item.html?id=94f838a535334cf1aa061846514b77c7
	Main Road speed	35km/hr	https://www.tomtom.com/en_gb/trafficindex/city/copenhagen
	Minor Road speed	30km/hr	
			https://books.google.co.nz/books?id=SBCmiaRdoGEC&pg=PT28&dq=copenhagen+traffic+congestion&hl=en&sa=X&ved=0ahUKEwj57syB4pXiAhUw6XMBHZlwAKEQ6AEINjAC#v=onepage&q=copenhagen%20traffic%20congestion&f=false
	General speed	Speed limits	
			https://books.google.co.nz/books?id=ZY7qISU8ZdIC&pg=PA87&dq=copenhagen+traffic+congestion&hl=en&sa=X&ved=0ahUKEwj57syB4pXiAhUw6XMBHZlwAKEQ6AEIPDAD#v=onepage&q=copenhagen%20traffic%20congestion&f=false
			https://en.wikipedia.org/wiki/Transport_in_Copenhagen
	All PT timetables		https://stateofgreen.com/en/publications/sustainable-urban-transportation/
			http://www.trafikdage.dk/td/papers/papers06/Trafikdage-2006-556.pdf
